# Plectoneme tip bubbles: Coupled denaturation and writhing in supercoiled DNA

**DOI:** 10.1038/srep07655

**Published:** 2015-01-07

**Authors:** Christian Matek, Thomas E. Ouldridge, Jonathan P. K. Doye, Ard A. Louis

**Affiliations:** 1 Rudolf Peierls Centre for Theoretical Physics, University of Oxford, 1 Keble Road, Oxford, OX1 3NP, United Kingdom; 2 Physical and Theoretical Chemistry Laboratory, Department of Chemistry, University of Oxford, South Parks Road, Oxford, OX1 3QZ, United Kingdom

## Abstract

We predict a novel conformational regime for DNA, where denaturation bubbles form at the tips of plectonemes, and study its properties using coarse-grained simulations. For negative supercoiling, this regime lies between bubble-dominated and plectoneme-dominated phases, and explains the broad transition between the two observed in experiment. Tip bubbles cause localisation of plectonemes within thermodynamically weaker AT-rich sequences, and can greatly suppress plectoneme diffusion by a pinning mechanism. They occur for supercoiling densities and forces that are typically encountered for DNA *in vivo*, and may be exploited for biological control of genomic processes.

Biological information is not only stored in the digital chemical sequence of DNA, but also in the mechanical properties of its strands, which can inuence biochemical processes involving its readout^1^. For example, loop formation in the Lac operon^2^ regulates the expression of key genes, and DNA supercoiling is correlated with circadian rhythms in cyanobacteria^3^. Supercoiling is also important for large scale organisation of the genome in both eukaryotic and prokaryotic cells. DNA can respond to torsional stress by writhing to form looped plectoneme structures^4^, thus transferring energy stored as twist into energy stored in bending. It can also form denaturation bubbles which absorb undertwist at the cost of breaking bonds^5^. The rich mechanical properties of DNA have been intensively studied by single-molecule techniques such as magnetic and optical tweezers^4^,^6^,^7^,^8^,^9^,^10^,^11^,^12^,^13^,^14^, and by theoretical techniques ranging from continuum models of DNA to atomistic simulations^5^,^10^,^15^,^16^,^17^,^18^,^19^,^20^,^21^.

Here, we study the interplay between plectonemes and bubbles using a recently developed model, oxDNA^22^,^23^, that treats nucleotides as rigid bodies with three interaction sites that mediate physically motivated attractive and repulsive interactions. OxDNA's resolution allows us, in contrast to continuum models, to study denaturations within supercoiled DNA. The model is also simple enough to explore the time and length scales relevant to plectonemes, something currently beyond the purview of atomistic simulations. OxDNA has successfully captured a number of systems in which base pairs break and form, including nanotechnological devices^24^ and biophysical processes such as overstretching^25^ and cruciform formation^26^, suggesting it is well suited for studying writhing and denaturation in supercoiled DNA.

First, we test oxDNA by calculating canonical “hat-curves” for strand extension over a range of applied torsions and forces similar to those used in single molecule assays^4^,^6^,^7^,^8^,^9^,^10^,^11^,^12^,^13^,^14^ and found *in vivo*. Torsion is quantified using the length-independent superhelical density *σ*; *σ* = +(−)1 for one full positive (negative) imposed turn per pitch length. We study a 600 base-pair (bp) duplex using an “average-base” parameterisation derived at 500 mM NaCl^22^,^23^. In this parameterisation, only AT and GC Watson-Crick base pairs can form, but interaction energies are set to average values independent of base identity. Ignoring sequence-dependence allows us to focus first on generic DNA behaviour^22^. Simulations are for one salt concentration, 500 mM NaCl, and further details are described in Methods.

Fig. 1a shows that, at a fixed force, the extension *L* (here normalised by the maximum extension *L*_0_) does not change appreciably as *σ* is increased from zero until a buckling transition occurs to a writhed plectonemic structure at *σ* = *σ_b_*(*F*). As *σ* is increased beyond *σ_b_*(*F*), the overall extension decreases linearly with *σ* because the extra writhe is stored in the growing plectoneme. For 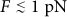 DNA strand extension is symmetric under *σ* → −*σ*. For 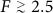 pN however, increasing negative *σ* causes the formation of bubbles, leading to little or no shortening of the strand. In all simulations, we observed at most one plectoneme, as is expected at high salt concentration and short strand length^12^,^17^. We show in SM Section II that oxDNA can accurately reproduce experimental measurements of hat curves and torque response^9^,^11^,^13^,^14^, giving confidence in the ability of the model to predict the fundamental features of DNA under torsion and tension.

We now address the unresolved question of how the system transitions between the plectoneme-dominated and bubble-dominated regimes. Instead of a simple transition between the two types of conformations, we observe an intermediate “tip-bubble” regime dominated by states with a co-localised bubble/plectoneme pair. This novel regime can be seen in the population diagram Fig. 1b, and in our overall schematic state-diagram shown in Fig. 1c. We now explore these tip-bubble states in more detail.

At the plectoneme tip, the duplex axis inverts its direction. For low forces, this is achieved by homogeneous bending. However, when curvature becomes larger an alternative is for bending to be localised at a kink defect involving a few broken base pairs, as recognised in studies on DNA bending^28^. Similarly, as the force is increased and the plectoneme tip becomes more tightly wound, a transition to a tip-bubble state can occur, as illustrated in Fig. 2a. Importantly, the sharper bending at the kink allows the same amount of writhe to be achieved by a smaller plectoneme, and thus the tip-bubble state is stabilised by an increase in the extension Δ*L* along the force. For negative supercoiling the tip bubble is also able to absorb some of the negative twist, allowing the plectoneme to shrink further. The latter is the reason why, although tip bubble formation is seen for positive and negative supercoiling (Fig. 1c) the transition occurs at a significantly higher force for positive *σ*.

For negative supercoiling and small forces around 1.0–1.5 pN, end-loop kinks are stable against further bubble growth because larger bubbles lead to a smaller contraction in plectoneme size per disrupted base pair than the initial kink, as shown in Fig. 2b. Therefore, further shrinking of the plectoneme does not compensate for the breaking of additional bonds. At larger forces, the tip bubble can grow to eliminate the plectoneme.

Fig. 2c shows a free-energy landscape in the vicinity of the transition from the tip-bubble regime to the bubble dominated regime. The landscape is relatively flat along the diagonal, showing that plectoneme size and bubble size can be easily interchanged, and that states with both bubbles and plectonemes of intermediate size are common. This tendency is even more evident in simulations of a larger plectoneme (see SM Section III).

It is instructive to compare this tip-bubble scenario to what might be expected if bubbles and plectonemes did not co-localize. The free-energy landscape would then be bi-stable, with plectoneme and bubble states separated by a substantial free-energy barrier due to the significant nucleation costs of both^5^,^9^,^12^. Such a scenario would lead to a sharp transition as tension is increased. Previous work that attempted to model the transition as a competition between bubble-dominated and plectoneme-dominated regimes^13^,^14^,^29^ has generally neglected the substantial nucleation costs of plectonemes and bubbles. Including these contributions would make the theoretical transitions much sharper than those observed in experiment. Due to the presence of tip bubbles, however, we find that each state reduces the nucleation cost of the other; namely, bubble growth occurs from the plectoneme tip, and the enhanced local bending flexibility of a bubble allows the DNA to more easily writhe. This scenario leads to a significantly broader transition, in better agreement with available experimental data (see also SM section III).

Fig. 2d depicts plectoneme diffusion kymographs. When there is no tip bubble, the plectoneme diffuses by reptation of the DNA through the plectonemic structure. But when posessing a tip bubble, plectonemes are effectively pinned because diffusion requires the coupled motion of the plectoneme and the writhed bubble at its tip. In the regime shown in the upper panel of Fig. 2d, tip bubbles form about 34% of the time, while being almost always present in the case shown in the lower panel.

To further quantify the dynamics of plectonemes we calculated their diffusion coefficients (see also SM Section VI). Fig. 3a shows diffusion coefficients for positive supercoiling. It is expected that diffusion via reptation will slow down with increasing tension^30^, an effect we observe by plotting the diffusion coefficient for configurations with no tip bubble. However, Fig. 3b shows that the full diffusion coefficient *D*_eff_ exhibits a marked further decrease as the fraction of time that the system is pinned increases. A reduction in plectoneme diffusion with force for positive supercoiling was observed in Ref. 12, although the experimental conditions do not allow a direct comparison. We discuss these results in SM Section VI.

Bubble formation is highly sequence dependent^5^,^31^. To explore the role of sequence, we performed additional simulations at *σ* = −0.06 and *F* = 1.27 pN using a sequence-dependent parametrisation of oxDNA for a fully random sequence with a GC content of 49%, and a block-random sequence containing five 120 bp stretches with a GC content alternating between 70% and 30% (average GC content of 52%; see SM Section VIII for sequences). Even though the average GC content is similar, tip bubble prevalence is 59% for the randomised sequence and 72% for the block-random sequence, compared to 19% for the average-base model. These differences occur because bubbles can form more easily in weaker AT-rich regions. This effect is also manifest in the hat-curves. The random sequence deviates from the *σ* → −*σ* symmetry at lower forces than the average-base model does (see SM Section VIII).

Fig. 3c shows the distribution of plectoneme locations for the random and block-random cases. Unlike the average-base model (see SM Section VIII) both sequences show strong localisation of plectonemes within AT-rich regions of the strand. Denaturations in tip-bubble plectonemes possess an average AT-content of 84% and 91% for the random and block-random sequences respectively. Hence through tip bubbles, local sequence properties can inuence the large-scale structure of DNA. At a typical negative *σ* found *in vivo*^1^, we estimate that tip bubbles will occur at 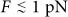 for physiological conditions (SM section II), a force regime common in living cells. We speculate that plectoneme localisation can be used by the cell to regulate access to weak parts of the sequence, which are known to be important in key biological processes, including transcription and replication^31^. In this context it is interesting to note that RNA polymerase, an enzyme that binds to the disrupted duplex, localizes at plectoneme tips^32^; this would be expected if duplex disruption is enhanced at these points.

In conclusion, we predict a conformational regime for DNA where plectonemes and denaturation bubbles are co-localised. This behaviour follows from the basic physics of a semi-flexible polymer with inherent chirality combined with nucleated denaturation and plectoneme formation. These are generic features of DNA under a range of conditions, so our results are likely to be qualitatively robust. One consequence of the tip-bubble state is a broad coexistence regime, which has already been observed experimentally^13^,^14^,^29^. Others are the accumulation of plectonemes in AT-rich parts of the DNA sequence, and a slowdown of their diffusive movement. Experimental observation of plectoneme positioning in AT-rich regions may be achieved with recently developed tools for the spatial resolution of plectonemes^12^, while direct observation of co-localisation of plectonemes and bubbles may be possible by combining these tools with reporter molecules to detect denaturation.

## Methods

Simulations were performed using an Andersen-like thermostat^27^ at 300 K. Trajectories were generated using a time step of 12.1 fs, and production runs were started from pre-thermalized configurations and run for 5 × 10^8^ time steps. During simulations, *σ* was fixed to values in the range −0.1 ≤ *σ* ≤ +0.1 by trapping the strand ends. Torsionally relaxed states were identified by demanding vanishing torque Γ on the traps. A force *F* with 0.25 pN ≤ *F* ≤ 7.9 pN was applied to the molecule ends; further details of the simulations are given in Section I of the Supplementary Material (SM).

## Supplementary Material

Supplementary Information Supplementary Movie: Plectoneme dynamics

Supplementary Information Supplementary Information

## Figures and Tables

**Figure 1:**
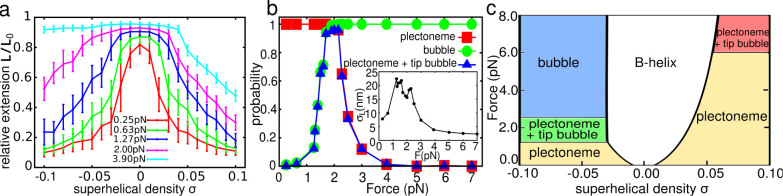
Double strand response to torsion and tension: (a), “Hat-curves” show the mean relative extension of a 600-bp duplex against superhelical density *σ* for various applied forces *F*. Error bars indicate thermal fluctuations in the end-to-end distance, rather than sampling uncertainties. (b), Mean fraction of plectonemes, bubbles and tip-bubble plectonemes, as a function of force for *σ* = −0.08. Inset: Fluctuations (standard deviation *σ_L_*) of end-to-end distances as a function of force for *σ* = −0.08 show two maxima, the first at the point when tip bubbles form in plectonemes, the second at the transition from tip-bubble plectonemes to bubbles only. Results for *L* = 1500 bp as well as for other values of *σ* can be found in Supplementary Material (SM) Sections III and VII. (c), State diagram of structures. Tip-bubble regions indicate points with at least a 40% probability of a plectoneme with a tip bubble.

**Figure 2:**
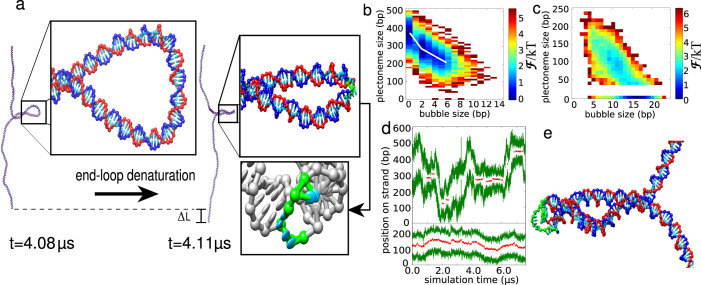
Plectoneme tip-bubble regime: (a), The 600-bp plectoneme system for *σ* = −0.05 and *F* = 1.27 pN. Enlarged structures show the end loops with and without denatured bases (coloured green). The formation of a 3-bp tip bubble leads to a smaller plectoneme, because the tip can bend more easily and absorb extra undertwist, leading to an increased extension of the full strand by Δ*L*. (b), A free-energy landscape for *σ* = −0.08 and *F* = 1.5 pN. The white line schematically shows the variation of the most likely plectoneme size with bubble size, illustrating the initial size reduction due to end-loop kinking. (c), A free-energy landscape for *σ* = −0.08 and *F* = 2.3 pN illustrating how the growth of bubbles leads to shrinking of the plectoneme. Tip-bubble plectonemes with small size (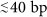) are hard to detect or distinguish from writhed bubbles and so are classed here as bubbles. See SM Section V for further information on the free energy landscape of tip bubbles. (d), Plectoneme kinetics depicted by kymographs of the plectoneme boundaries (green lines). Red denotes the centre of denatured base-pair stretches (bubbles), which pin the plectoneme and slow diffusion. The upper panel shows a simulation at *σ* = −0.05 and *F* = 1.27 pN, from which the structures in (a) are taken. The lower panel shows a simulation for a fully pinned state at positive supercoiling, *σ* = +0.08, *F* = 7.9 pN, exhibiting much slower effective diffusion. (e), Structure of a tip-bubble plectoneme at *σ* = −0.08 and *F* = 2.3 pN, posessing a 12-bp tip bubble and a 134-bp plectoneme. Denatured nucleotides are coloured green.

**Figure 3:**
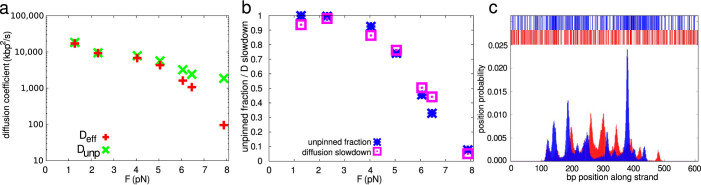
Plectoneme diffusion and sequence-dependent localization: (a), Diffusion coefficients for plectonemes at positive *σ* as a function of stretching force. *σ* is chosen as in Ref. 12 such that approximately 25% of the strand length is in the plectonemic state on average. The diffusion constant *D*_unp_ for unpinned plectonemes (green) is compared to the observed effective diffusion constant *D*_eff_ (red). (b), Fraction of time plectonemes are found without a tip bubble (blue) compared to the relative slowdown of diffusion *D*_eff_/*D*_unp_ (magenta). The close agreement suggests that the slowing down of the observed *D*_eff_ compared to *D*_unp_ is mainly due to pinning. (c), Position distribution of plectonemes at *σ* = −0.06 and *F* = 1.27 pN. Results are shown in red for a random sequence, and in blue for a block-random sequence, as explained in the text. Plectoneme formation is suppressed near strand ends because these are clamped. For each sequence, the upper part of the figure shows coloured positions for AT basepairs and white for CG basepairs. Plectonemes in the tip-bubble regime strongly localize to AT-rich regions.
