# Supplementary material for: Plectoneme tip bubbles: Coupled denaturation and writhing in supercoiled DNA
Source: Sci Rep. 2015 Jan 7;5:7655. doi: 10.1038/srep07655 (PMC5224516; doi:10.1038/srep07655)
Supplement: Supplementary Information [file srep07655-s2.pdf]

Plectoneme tip bubbles: Coupled denaturation and writhing in supercoiled DNA
Supplementary Material

Christian Matek,[1] Thomas E. Ouldridge,[1] Jonathan P. K. Doye,[2] and Ard A. Louis[1,*]

[1]*Rudolf Peierls Centre for Theoretical Physics, University of Oxford, 1 Keble Road, Oxford, OX1 3NP, United Kingdom*

[2]*Physical and Theoretical Chemistry Laboratory, Department of Chemistry, University of Oxford, South Parks Road, Oxford, OX1 3QZ, United Kingdom*

[*]Correspondence and requests for materials should be addressed to:ard.louis@physics.ox.ac.uk

I. SIMULATION METHODS AND BOUNDARY CONDITIONS

Simulation algorithm

As oxDNA is an implicit solvent model, it is inappropriate to directly integrate the equations of motion resulting from its interaction potentials, which would lead to ballistic particle motion. Instead, we used the Andersen-like thermostat that is described in more detail in Ref. 1. It propagates the system for a set number of time steps N_{Newt} according to Newton's equations, using the common Verlet integrator with a time step of 12.2 fs. Linear and angular velocities in the system are then assigned new values drawn from a Maxwell-Boltzmann distribution at $T = 300\,\mathrm{K}$ with probabilities p_{lin} and p_{ang}. For all simulations performed for this work, we chose $N_{\mathrm{Newt}} = 103$, $p_{\mathrm{lin}} = 0.0204$ and $p_{\mathrm{ang}} = 0.0068$. The time-scales reported in this paper were set by mass, energy and length scales used in the integrator. However, making direct comparisons of time-scales between coarse-grained simulations and experiments is complex, see for example the discussions in Refs. 2–4, and in Supplementary Sec. VI, where we discuss diffusion. Similar simulations settings have been successfully used in other applications of oxDNA (see e.g. Refs. [5–7]).

Boundary conditions

In this work, we study the response of DNA to superhelical stress. A common measure of superhelical stress is the linking number Lk, a topological quantity which equals the number of times the single strands wrap around each other. In topologically constrained systems, Lk decomposes according to the Fuller-White-Călugăreanu relation $Lk = Tw + Wr$ [8–10], where Tw is the number of single-strand crossings, and Wr the number of self-crossings of the double strand axis of the system. To facilitate comparison to systems of different strand length, we quantify torsion using the length independent superhelical density defined as $\sigma = (Lk - Lk_0)/Lk_0$, where Lk_0 is the number of single-strand crossings in a torsionally relaxed, linear double strand.

To impose superhelical stress in a simulation, we constrain the ends of a double strand, and disallow passing of the double strand across the strand ends. Similar constraining boundary conditions have been successfully used before in simulations of cruciform extrusion [11]. A schematic overview of the boundary conditions applied is shown in Fig. S1.

Fixation is implemented by adding five boundary base pairs to the strand ends, which are trapped in harmonic potentials. These potentials acting on the n-th trapped nucleotide have the form

$$V_{\mathrm{trap}}(\mathbf{r}_n; \mathbf{r}_{n,0}) = \frac{1}{2} \sum_{i=1}^{3} k^i_{\mathrm{trap}} (r^i_n - r^i_{n,0})^2, \tag{1}$$

where $\mathbf{r}_n = (r^1_n, r^2_n, r^3_n)$ is the centre-of-mass position of the n-th trapped nucleotide and the corresponding trap position is $\mathbf{r}_{n,0} = (r^1_{n,0}, r^2_{n,0}, r^3_{n,0})$, chosen initially such as to fix a given twist angle of the strand. To keep the overall twist angle on the system fixed, but ensure free extensibility of the strand along the setup axis $\hat{\mathbf{x}}_3$, we choose $k^{\mathrm{trap}}_1 = k^{\mathrm{trap}}_2 = 57.1\mathrm{N/m}$ and $k^{\mathrm{trap}}_3 = 0$. The traps defined in this way only constrain the end nucleotides in a co-moving 2-dimensional plane perpendicular to the strand setup axis, while not hindering the strand in the $\hat{\mathbf{x}}_3$ direction. High trap stiffness in that plane ensures that fluctuations of the linking number Lk in the course of a simulation are negligible.

Strands have finite lengths, which means that more distant parts of the system can pass around the strand ends. Such a process would change the linking number Lk and thus the superhelical density σ of the system. We therefore prevent this process by introducing repulsion planes oriented perpendicular to the setup axis $\hat{\mathbf{x}}_3$ which co-move with the first boundary nucleotide of the two single strands in the system. Repulsion planes generate a potential

$$V_{\mathrm{plane}}(\mathbf{r}; \mathbf{R}) = \frac{1}{2} k^{\mathrm{plane}} \left((\mathbf{r} - \mathbf{R}) \cdot \hat{\mathbf{o}}\right)^2 \theta(-(\mathbf{r} - \mathbf{R}) \cdot \hat{\mathbf{o}}), \tag{2}$$

where $\mathbf{r}$ is the centre-of-mass position of an affected particle, $\mathbf{R}$ and $\hat{\mathbf{o}}$ are anchor point and orientation of the plane, and θ is the Heaviside step function. We choose $\hat{\mathbf{o}} = \hat{\mathbf{x}}_3$ and $\hat{\mathbf{o}} = -\hat{\mathbf{x}}_3$ for the lower and upper repulsion planes respectively, and set $\mathbf{R}$ to the instantaneous positions of the the first and last double strand boundary base pair. To avoid hindering free strand extensibility in the $\hat{\mathbf{x}}_3$ direction, the repulsion

planes do not interact with the next-to-last boundary base pairs at both strand ends. In all simulations, we chose $k^{\text{plane}} = 28.5\,\text{pN/nm}$, which prevented the duplex from passing over its ends.

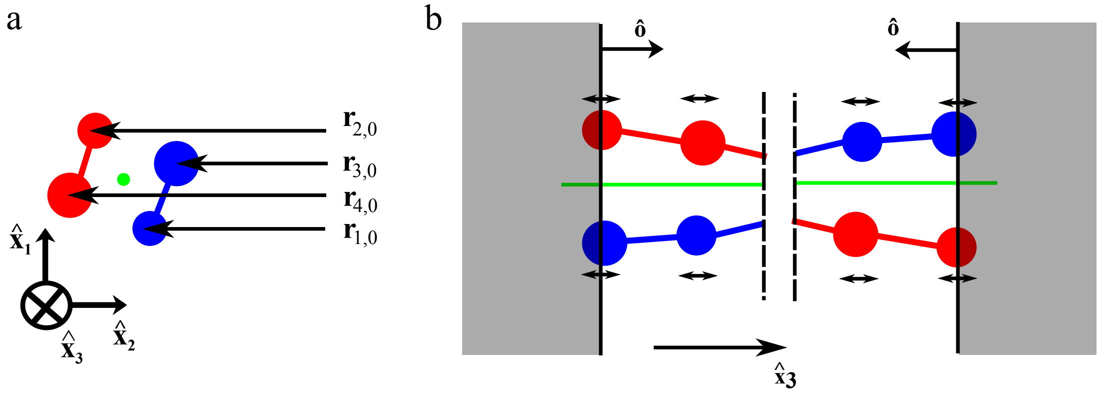

FIG. S1. Schematic depiction of the applied boundary conditions illustrated for the last 2 bp at each end of the strand: (a) View along the strand axis. 5 nucleotides at each strand end are constrained by 2-dimensional harmonic traps, which fix boundary nucleotides to positions $\mathbf{r}_{n,0}$ in planes perpendicular to the strand axis (green). (b) View perpendicular to the double strand. Due to the 2-dimensional traps, nucleotides are unconstrained only in the strand-axis direction. A repulsion plane perpendicular to the strand axis is tagged to the last base pair. Movement of nucleotides into the area below the end base pair (shaded grey) is excluded. In order to allow unconstrained strand extensibility, the repulsion plane does not act on the first two base pairs along the strand.

Microscopic determination of base-pair breaking

In several sections of this work, we determine if a given base pair is formed or broken. As in previous applications of oxDNA [12, 13], a base-pair was counted as formed if the energy contribution from hydrogen-bonding was below -4.13×10^{-21} J, corresponding to approximately 15% of the typical energy of a fully formed hydrogen bond.

Sequences and symmetry of hydrogen bonding

The double strands studied in this work possess fully complementary sequences. Hydrogen bonding was only permitted between each nucleotide and its direct counterpart on the other single strand, excluding shifted bonds as shown in Fig. S2. This restriction was imposed to avoid non-generic secondary structure effects, and increase simulation efficiency. It has been used successfully before for other studies with oxDNA [5–7, 11]. The sequences used within the sequence-dependent parametrization are given in Supplementary Sec. VIII.

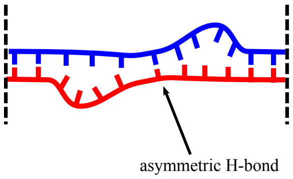

FIG. S2. Asymmetric hydrogen bonds were excluded in simulations.

II. COMPARISON OF MECHANICAL BEHAVIOUR TO EXPERIMENTAL DATA

Framework for comparison

The mechanical behaviour we observe can be compared directly to experimental data from single molecule assays. Most available data has been obtained using magnetic [14],[15],[16],[17] or optical tweezers [18], which can measure the end-to-end extension and torque response of a single DNA molecule. Available datasets differ in molecule length L, ionic strength of the buffer, and sequences properties of the DNA strands used in the respective experiments.

Here, we relate our results to experimental findings and explain the influence of different parameters on the quality of agreement. We note that oxDNA is limited to an ionic strength of $[\mathrm{Na}^+] = 500\,\mathrm{mM}$, as the effective potentials of the model were parametrized at that salt concentration. Furthermore, for reasons of computational efficiency, we simulated double strands of length $L = 600\,\mathrm{bp}$, for which equilibrium data for end-to-end extension can be obtained within about two weeks of runtime on a current CPU. This is shorter than the strand lengths typically used in experiment, which will affect the comparison of results.

In order to facilitate comparison to experimental data, we report our results in terms of the relative strand extension $l = L/L_0$ and the superhelical density σ, which are length-independent up to finite-size corrections vanishing in the long-strand limit [19]. Some uncertainty exists about the relaxed length L_0, because in typical experimental assays, measurements of end-to-end distance take place on molecules which are attached to a cover slip and a bead via chemically functionalized ends. Attachment may slightly modify the effective free length of the double strand. The simulated system only uses a very short piece of strand to constrain the duplex ends (see Supplementary Sec. I) and therefore does not include these chemical attachment effects. Uncertainty about the effective free length of attachment may therefore introduce small systematic differences when comparing experiments to our simulations, especially when measuring the absolute length L. By contrast, experimental measurements of the buckling superhelical densities σ_b and postbuckling slopes $dl/d\sigma$ should be largely unaffected by the length of attachment, and may therefore be easier to compare with our simulations.

The buckling superhelical density σ_b is determined primarily by the values of bending persistence length and twist persistence length [15], which in oxDNA take the values $B_0 = 42.5$ nm and $C_0 = 114.7$ nm respectively [12]. As these values lie in the range of values reported for DNA [17], oxDNA might be expected to reproduce experimental values for σ_b. Differences with experiment may simply stem from small differences in the value of these constants, and would occur for any elastic model using these values for B_0 and C_0.

The post-buckling slopes are thought to mainly depend on the radius of the plectoneme stem, which is set by the physics of twisting and bending, as well as by screened electrostatic interactions between the double strands [20, 21]. Good agreement of post-buckling slopes may thus be an indication for the consistency of oxDNA's treatment of strongly screened electrostatics by excluded volume interactions.

In addition to reproducing elastic properties of the double strand, oxDNA provides a good representation of the melting curves of DNA, as well as several other systems where breaking of base pairs plays a role [5, 7, 12]. This gives us confidence to apply the model to study plectonemes, in which both effects of strand elasticity and double strand denaturation might be expected to be relevant.

All simulation results presented in this section were obtained using the average-base parametrization of oxDNA. Details of the mechanical response of DNA to imposed twist may however depend on the specific sequence used. In particular, the precise value of the crossover force F_{char} between bubbles and plectonemic structures might be expected to depend on sequence properties of the DNA strand, as the enthalpic cost of bubble formation decreases with increasing AT content. Therefore, AT-rich stretches represent preferred nucleation sites for denaturation bubbles, which can facilitate denaturation and therefore decrease F_{char}. The effect of different sequences is described in more detail in Supplementary Sec. VIII.

Molecular extension "hat curves"

Measurements of strand extension as a function of applied force F and superhelical density σ are very reproducible, and have been measured in many setups for different ambient conditions [14–17, 22, 23]. Here, we compare to a set of experiments performed at conditions close to those used in the parametrization of oxDNA. Our strand extension results are plotted together with experimental data in Figs. S3 - S5.

Fig. S3 shows a comparison to experimental data of Tempestini *et al.* [22] on a stand of length 7 kbp, with an effective free length of 5.8 kbp. This effective length is inferred from the length of the attachment described in Ref. 22. The salt concentration used in these experiments is $[\mathrm{Na}^+] = 500\,\mathrm{mM}$, the value at which oxDNA was parametrized. Post-buckling slopes are in good agreement with simulation data, whereas buckling occurs at slightly lower experimental superhelical density σ_b. This may be due to additive finite-size corrections $O(L^{-1/2})$ predicted in continuum models of the buckling transition [19].

Fig. S4 shows a comparison to data by Salerno *et al.* [23], obtained at $L = 6\,\mathrm{kbp}$ and a lower salt concentration $[\mathrm{Na}^+] = 150\,\mathrm{mM}$. Lower salt is expected to destabilize plectonemes and make bubbles more favourable, as electrostatic interactions of the backbone are less screened. In particular, this effect is expected to become more significant for $\sigma < 0$, as has been shown experimentally [22]. Comparing to simulation results, we still observe good agreement in the post-buckling slopes for all forces when $\sigma > 0$. In contrast, the crossover to the extended bubble state for $\sigma < 0$ happens at higher forces for oxDNA. That the force is higher is expected because of the difference in the salt concentration between simulation and experiment. Although appropriate hat curves are unavailable at higher salt for $\sigma < 0$, data on the fluctuation of end-to-end lengths from Ref. 22 suggests that the transition to the extended bubble state occurs at roughly 1 pN, about 1 pN below our estimate for $[\mathrm{Na}^+] = 500\,\mathrm{mM}$. We discuss these differences in more detail in Supplementary Sec. III on end-to-end fluctuations, and Supplementary Sec. VIII on sequence dependence, where we show that since AT rich regions are more likely to form bubbles, taking this into account with a sequence dependent model leads to lower forces for the crossover to bubbles than using an average base model for oxDNA does.

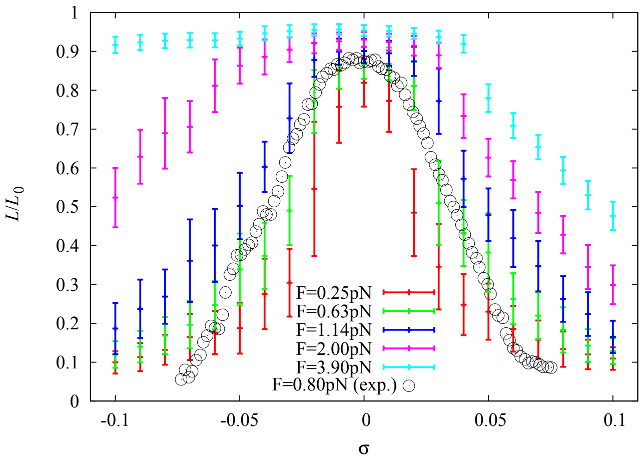

FIG. S3. Experimental strand extension data of Ref. 22 at $[\mathrm{Na}^+] = 500\,\mathrm{mM}$ and $L \approx 5.8\,\mathrm{kbp}$ for $F = 0.8\,\mathrm{pN}$, compared to data from simulations using oxDNA ($[\mathrm{Na}^+] = 500\,\mathrm{mM}$ and $L = 600\,\mathrm{bp}$). Error bars on simulation results indicate thermal fluctuations in the end-to-end distance, rather than sampling uncertainties. Good agreement is observed, while nonlinear effects in the post-buckling slopes for high $|\sigma|$, caused by end effects, are somewhat more pronounced for the shorter simulated system.

In the data of Refs. 23 and 22, deviations of the post-buckling slopes from linearity were observed at low stretching forces and high values of $|\sigma|$. Similar, more pronounced deviations are observed in our results due to finite-size effects arising from the interaction of the coiled DNA strand with the system boundaries (cf. Supplementary Sec. I). Boundary interactions become relevant when the diameter of the plectonemic region is comparable to the end-to-end extension of the double strand. This is expected to be the case at somewhat lower values of $|\sigma|$ in the 600-bp system studied here, as compared to the roughly 10 times longer experimental systems of Refs. 23 and 22, shown in Figs. S3 and S4.

Fig. S5 depicts data obtained by Brutzer *et al.* [15] at $[\mathrm{Na}^+] = 320\,\mathrm{mM}$, $L = 1.9\,\mathrm{kbp}$ and $\sigma > 0$. Close

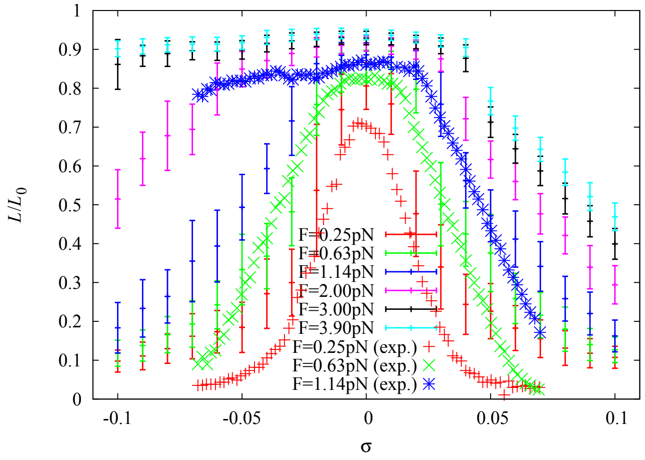

FIG. S4. Experimental strand extension data of Ref. 23 at $[\mathrm{Na}^+] = 150\,\mathrm{mM}$ and $L \approx 6\,\mathrm{kbp}$ for $F = 0.25\,\mathrm{pN}$, $0.63\,\mathrm{pN}$ and $1.14\,\mathrm{pN}$ compared to data from simulations using oxDNA ($[\mathrm{Na}^+] = 500\,\mathrm{mM}$ and $L = 600\,\mathrm{bp}$). Data for equal stretching force is represented by the same color. Error bars on simulation results indicate thermal fluctuations in the end-to-end distance, rather than sampling uncertainties. At lower salt concentration, plectoneme structures are enthalpically penalized as electrostatic interactions are screened less, making them less favourable compared to bubble configurations. Lower salt concentration also reduces the free-energy cost of base pair breaking. Hence, the crossover from tip-bubble plectonemes to extended bubble states occurs at lower forces than would be the case for $[\mathrm{Na}^+] = 500\,\mathrm{mM}$. Sequence dependence may additionally decrease the crossover force F_{char} by providing AT-rich bubble nucleation sites, as discussed in Supplementary Sec. VIII. Non-linear regions in the post-buckling slope are somewhat more pronounced for the shorter simulated system.

agreement is observed concerning both the buckling superhelical densities σ_b and the post-buckling slopes $dl/d\sigma$ (Fig. S5). We note that in this study, abrupt strand shortening at the buckling point was observed and ascribed to the energetic cost of forming the plectoneme end-loop. Abrupt strand shortening was shown to become more pronounced with increasing ionic strength. In oxDNA, we consistently observe a marked shortening of the strand at similar force around the buckling point (see Fig. S5; for an example with the sequence-dependent parametrization of oxDNA, see Fig. S26).

Torque response curves

The torque response of DNA to imposed twist has been studied for systems of different length and salt concentration [16–18]. A common feature of the torque response is a linear regime for low $|\sigma|$, followed by a torque overshoot at buckling and a constant post-buckling torque. The published dataset which is closest to the conditions used in oxDNA is the one recently measured by Janssen *et al.* [16] at $[\mathrm{Na}^+] = 550\,\mathrm{mM}$ and $L = 7.9\,\mathrm{kbp}$ using a variant of the magnetic tweezer setup. A comparison to the torque response data is shown in Fig. S6, exhibiting very good agreement for both the size and location of the torque overshoot, as well as the slope of the torque response in the linear regime and the magnitude of the constant post-buckling torque.

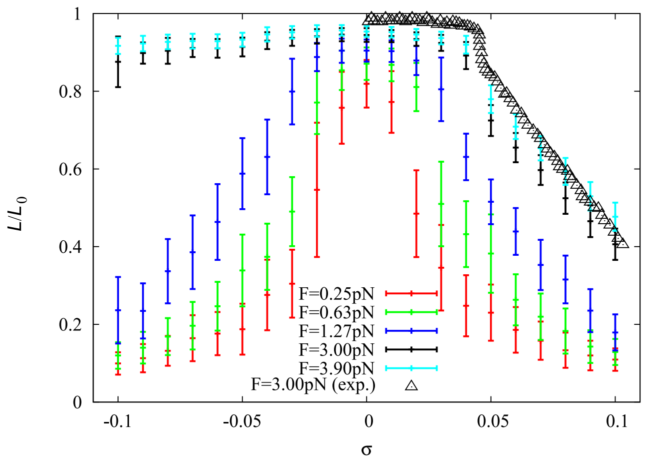

FIG. S5. Experimental strand extension data of Ref. 15 (black triangles) for $[Na^+] = 320\,mM$ and $L = 1.9\,kbp$ at $F = 3.0\,pN$ compared to data from simulations using oxDNA ($[Na^+] = 500\,mM$ and $L = 600\,bp$). For simulation data, error bars indicate standard deviations due to thermal fluctuations, rather than sampling uncertainties. Good agreement of buckling point and post-buckling slope is observed. Note also the abrupt length reduction upon buckling, present in both experiment and simulations.

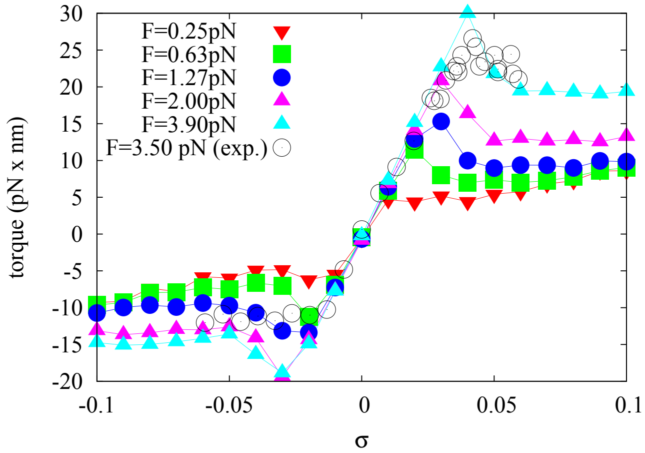

FIG. S6. Experimental torque response data of Ref. 16 at $[Na^+] = 550\,mM$ and $L = 7.9\,kbp$ for $F = 3.50\,pN$ compared to data from simulations using oxDNA ($[Na^+] = 500\,mM$ and $L = 600\,bp$). Excellent agreement is observed both in the linear regime (see also Fig. S7) and for the location and size of the torque overshoot.

Correspondence to Moroz-Nelson theory

The effective twist persistence length of a DNA strand is renormalized because of thermal fluctuations [24]. By using a torsional directed walk model, Moroz and Nelson estimated the effect of fluctuations to lead to an effective twist persistence length of

$$C_{\mathrm{eff}} = C_0 \left[1 - \frac{C_0}{4B_0} \sqrt{\frac{k_B T}{B_0 F}} \right] , \tag{3}$$

where B_0 and C_0 are the microscopic bending and twist persistence lengths of the double strand respectively. In oxDNA, these values have previously been determined as $B_0 = 42.5\,\mathrm{nm}$ and $C_0 = 114.7\,\mathrm{nm}$ [12]. We determined the force-dependent effective twist persistence length C_{eff} of the simulated 600-bp system by fitting the slope of the linear regime of the torque response curve (see e.g. Fig. S6). Good agreement of the measured torsional moduli with the theoretical prediction is observed (Fig. S7). If the functional form of Eq. 3 was fitted to the data with B_0 and C_0 as free parameters, we obtain $B_0 = 40.8\,\mathrm{nm}$ and $C_0 = 115.6\,\mathrm{nm}$, which are very similar to the previously determined values. Note that the agreement shown in Fig. S7 is obtained without free fit parameters.

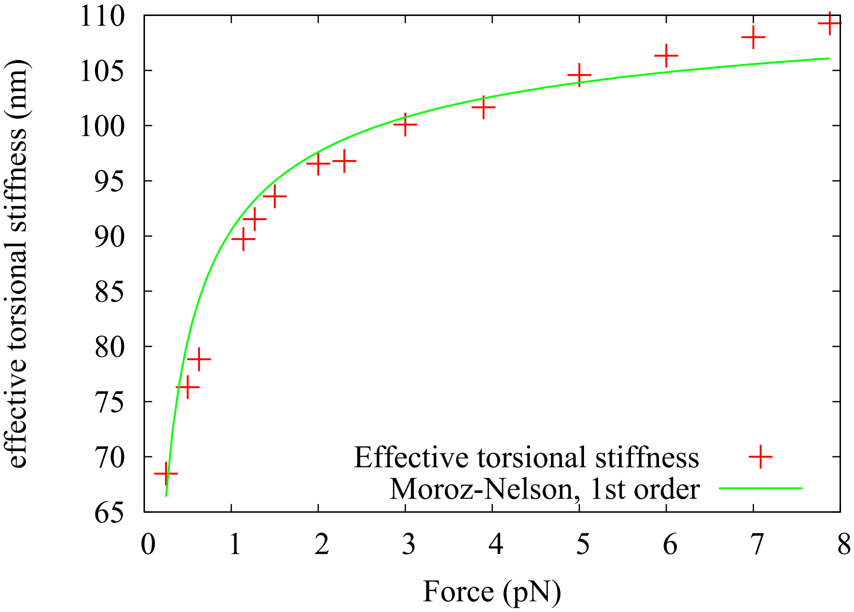

FIG. S7. Prediction of the effective twist persistence length from microscopic values according to Moroz-Nelson theory (green lines show first-order expansion; no free parameters) and values determined from simulations (red crosses).

Summary and modelling philosophy

In summary, oxDNA is generally consistent with the available experimental data. The shape of hat curves matches those seen in experiment; buckling super-helical densities, post-buckling slopes and torque response are quantitatively similar to the experimental data obtained under the most relevant conditions. For negative supercoiling at higher forces, systems form denaturation bubbles rather than plectonemes, as in experiment. From the data available, it seems that oxDNA with sequence-independent parameters might slightly overestimate the force at which this transition occurs in equivalent experimental conditions. The purpose of this work, however, is not to predict the exact location of certain transitions, which may be subject to a range of subtle influences. Rather, it is to explore the natural consequences of applying tension and twisting stress to a semi-flexible, inherently twisted polymer with the ability to undergo nucleated denaturation and plectoneme formation, with parameters that are broadly consistent with physical DNA. We find that combining these ingredients leads to a qualitatively novel prediction – bubbles and plectoneme tips will co-localize. This behaviour arises from the need to nucleate both plectonemes and bubbles, and the fact that bubbles can relieve stress at the tips of plectonemes whilst

allowing additional extension along the stretching force. Individually, these facts are well established [25, 26], and hence it is likely that our results are qualitatively robust.

As expected, oxDNA is most comparable on a quantitative level to experiments performed under high salt conditions. At lower salt, hat curves are known to be quantitatively different; buckling points and post-buckling slopes change, and the force required to induce denaturation with negative supercoiling is reduced. These changes are expected based on the reduced stability of duplexes, and the reduced ability to bend or wind duplexes tightly at lower salt concentrations when electrostatic repulsion is more relevant. Overall, we expect tip-bubble plectonemes to occur at lower forces for lower salt. However, the above description of DNA as a semi-flexible, inherently twisted polymer with the ability to undergo nucleated denaturation and plectoneme formation remains valid over a wide range of salt conditions. The root causes of the phenomena observed in this manuscript are still present, and thus qualitatively similar behaviour would be expected, even if the quantitative details vary.

III. SIMULATIONS OF PLECTONEMES OF LENGTH 1500 BP

For reasons of computational efficiency, the majority of the data presented in the main paper of this work was taken from systems at a strand length of $L = 600$ bp. In order to study the influence of differences in strand length and for comparison with experimental work we also performed simulations at strand length $L = 1500$ bp, for which a version of oxDNA for CUDA-enabled GPUs was used.

At forces between 1 and 3 pN, the 600-bp fluctuation spectrum exhibits enhanced fluctuations, and shows a two-peak substructure (see inset of Fig. 1b in the main text). The first peak may be explained by fluctuations arising from the initial opening of the tip bubble, while the second peak is due to the competition between a tip-bubble plectoneme and the extended bubble state. From this consideration, the first peak of the fluctuation spectrum might be expected to be approximately independent of system size, kink formation being a local effect associated with the plectoneme end-loop. On the other hand, the high-force peak of the enhanced fluctuation region is expected to grow linearly with system size for constant σ, as it is related to the plectoneme size, and the maximum size of a plectoneme is set by the absolute linking difference $\Delta Lk = \sigma(N-1)/p$, where N is the number of base pairs in the strand, and $p \approx 10.4$ is the average number of base pairs per turn.

To test this expectation, we ran simulations of the 1500-bp system at $\sigma = -0.08$ and $F = 1.5$ pN and $F = 2.3$ pN, near the expected peaks in the fluctuation spectrum. In particular the second point is difficult to sample, as it involves a global redistribution of ΔLk, which is known to be a relatively slow process [15]. We ran four independent simulations of a total simulation time of approximately 128 μs at $F = 1.5$ pN and ten independent simulations of a total simulation time of approximately 443 μs at $F = 2.3$ pN. The end-to-end distributions obtained for these state points at $L = 600$ bp and $L = 1500$ bp are shown in Figs. S9 and S10. At $F = 2.3$ pN, clear bimodal behaviour is observed, where a high-extension population represents the extended bubble state, and a low-extension population the tip-bubble plectoneme state. We note that a somewhat similar distribution has been reported from experiments in Ref. 15 for positive supercoiling, where the low-extension state is the unwrithed structure without bubbles and the high-extension case is expected to be plectonemes without tip-bubbles. In our case the low-extension population consists of plectonemes with tip bubbles and is much broader because plectonemes with tip bubbles of different sizes have comparable stabilities. We observe further substructure in the low-extension distribution for $L = 1500$ bp, but would caution that the sampling of large supercoiled structures converges slowly and so we have not been able to verify that this feature is robust. At any rate, we expect its influence on the standard deviation calculated for the distribution to be small.

Fluctuation results for both 600 bp and 1500 bp are plotted together in Fig. S8, corroborating the expectation that the first peak is largely independent of system size, while the second peak grows much more strongly, with an amplitude increase consistent with our expectation that it would grow roughly linearly with system size.

In a series of pioneering experiments [22, 23], the fluctuation spectrum in L/L_0 was measured for the first time for systems of effective length $L \approx 6$ kbp, around the region for negative supercoiling where there is a crossover from plectonemes to a bubble state. By extrapolating the increase in σ_L observed in our simulations, we would predict fluctuations to have a maximum magnitude of roughly 200 nm at the higher force peak which is consistent with what was observed in experiment. We note that these experiments probably could not resolve the first peak, as this is expected to remain very small. The overall width of the peak in the fluctuation spectrum observed in experiments is somewhat more narrow than it is in our simulations for $L = 600$. One reason is that the experiments would mainly resolve the higher force peak, for which the width at half maximum is smaller than the full spectrum we observe at this shorter length. A second reason is that generic finite size effects should lead to a narrowing of the fluctuation peak at a transition for increasing system size. Nevertheless, as also discussed in the main text, the experiments discussed above observed considerably wider peaks in the fluctuation spectrum than they predicted based on a simple model that only includes plectonemes and bubbles. We argue that the bubbles and tip-bubble plectonemes each lower the nucleation barrier for the formation of the other, leading to a broader fluctuation spectrum than one would observe for two states separated by a large nucleation barrier.

We also note that for a similar salt concentration, the experiments of Ref. 22 find the fluctuation peak at $F \approx 1$ pN, a position about one pN lower than our large force peak. There may be a number of reasons for this. Firstly, there are still unexplored generic finite size effects that may lower the transition force for longer strands. Secondly, simulations for both strand lengths reported here have been performed for the average base parameterisation of oxDNA. As discussed in Supplementary Sec. VIII, when using

a sequence-dependent model, the crossover between bubble and plectoneme states takes place at lower forces because bubbles preferentially form at AT rich regions. Moreover, for a random sequence, the longer the strand, the higher the probability for finding larger AT rich regions, which may also further enhance the probability of bubble formation. Taken together, these effects are expected to lower the forces at which the fluctuation peak occurs, bringing better agreement with the force at which a fluctuation maximum is observed in the experiments of Ref. 22. Finally, we note that it may also be the case that oxDNA overestimates the cost of forming twist-induced bubbles. It is hard to find direct comparisons to experiment for this phenomenon that would allow for an independent check. As previously mentioned, we we do find good agreement with experiment for force induced melting [5] or for simple duplex melting [12].

Finally, in Fig. S11, typical tip-bubble plectoneme configurations at $L = 1500\,\text{bp}$ observed for $F = 2.3\,\text{pN}$ and $\sigma = -0.08$ are shown. We note that these structures are remarkably long-lived once they form. A kymograph for a tip-bubble plectoneme in the $L = 1500\,\text{bp}$ system at $F = 2.3\,\text{pN}$ and $\sigma = -0.08$ is shown in Fig. S12, demonstrating that these structures can be stable on a μs timescale.

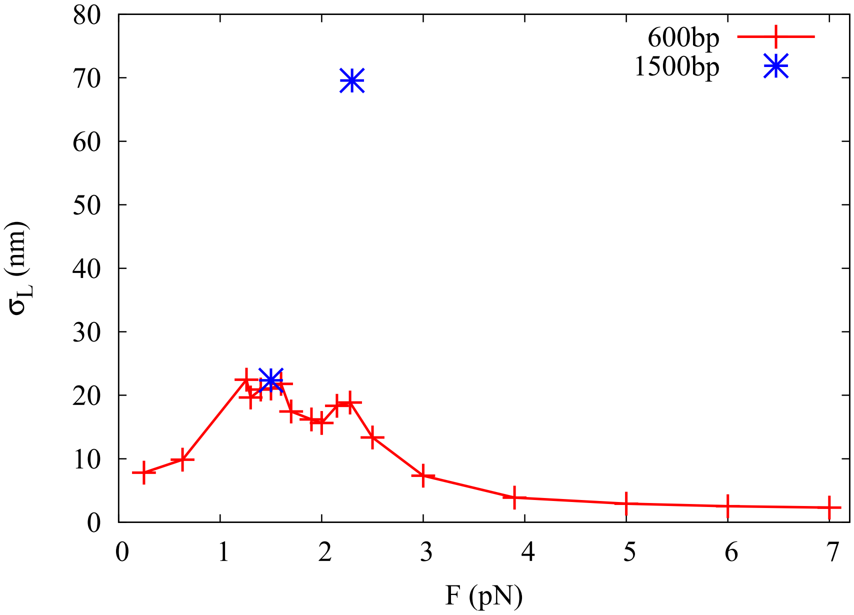

FIG. S8. Fluctuation spectrum (standard deviations of the end-to-end distance) for $L = 600\,\text{bp}$ (red points, as in inset of Fig. 1b in the main paper), together with two points obtained for a $L = 1500\,\text{bp}$ system (blue points). While the maximum at low force is approximately independent of strand length, the high-force maximum grows with system size in a roughly linear fashion.

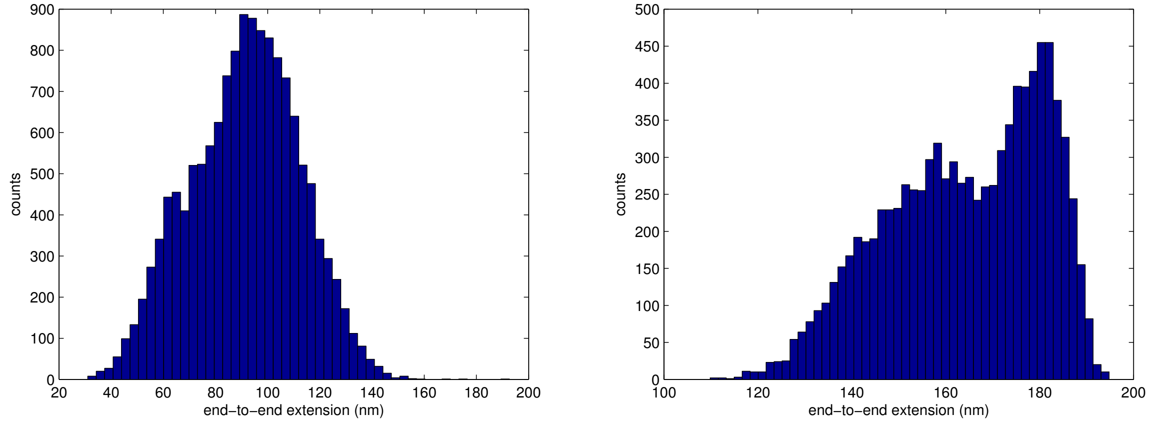

FIG. S9. Extension distributions for $L = 600\,\text{bp}$, at superhelical density $\sigma = -0.08$, with $F = 1.5\,\text{pN}$ (left) and $F = 2.3\,\text{pN}$ (right). Note the weak bimodality in the right histogram, due to interconversion between bubble and tip-bubble plectoneme states.

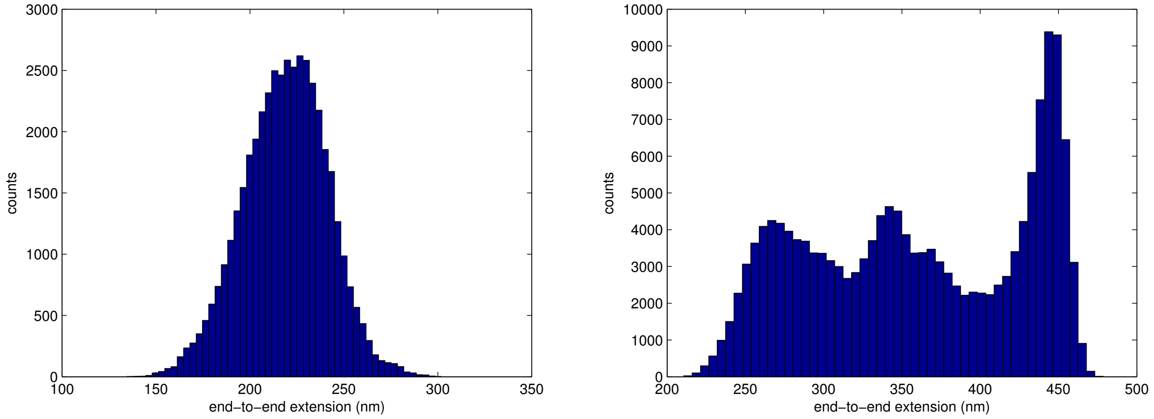

FIG. S10. Extension distributions for $L = 1500\,\text{bp}$, at superhelical density $\sigma = -0.08$, with $F = 1.5\,\text{pN}$ (left) and $F = 2.3\,\text{pN}$ (right). The results are qualitatively similar to those for the $L = 600\,\text{bp}$ system shown in Fig. S9.

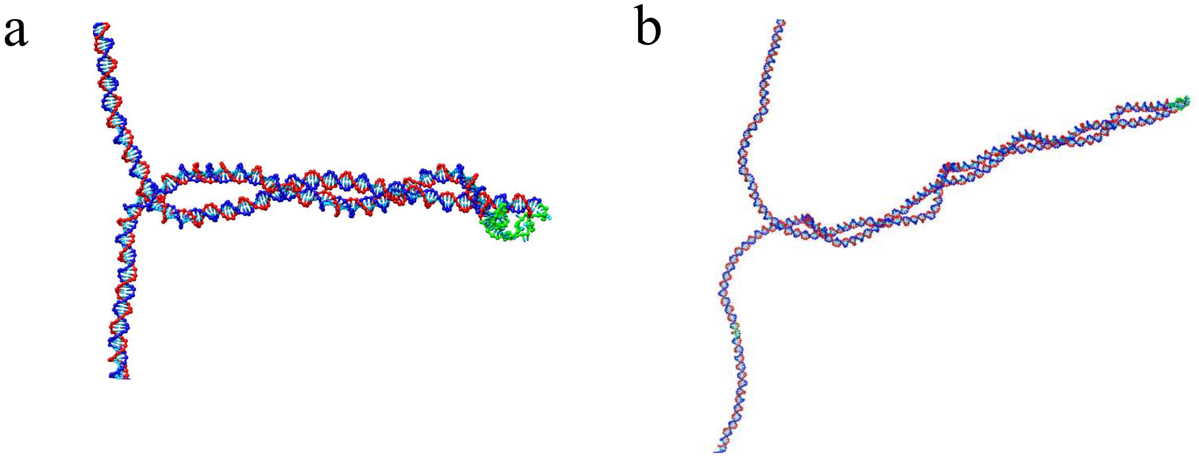

FIG. S11. (a) Tip-bubble plectoneme structure at $L = 1500\,\text{bp}$, for $\sigma = -0.08$ and $F = 2.3\,\text{pN}$, with plectoneme size 211 bp and bubble size 33 bp. (b) Tip-bubble plectoneme structure at $L = 1500\,\text{bp}$, for $\sigma = -0.08$ and $F = 2.3\,\text{pN}$, with plectoneme size 576 bp and bubble size 10 bp. Note that both structures are obtained at the same values of σ and F.

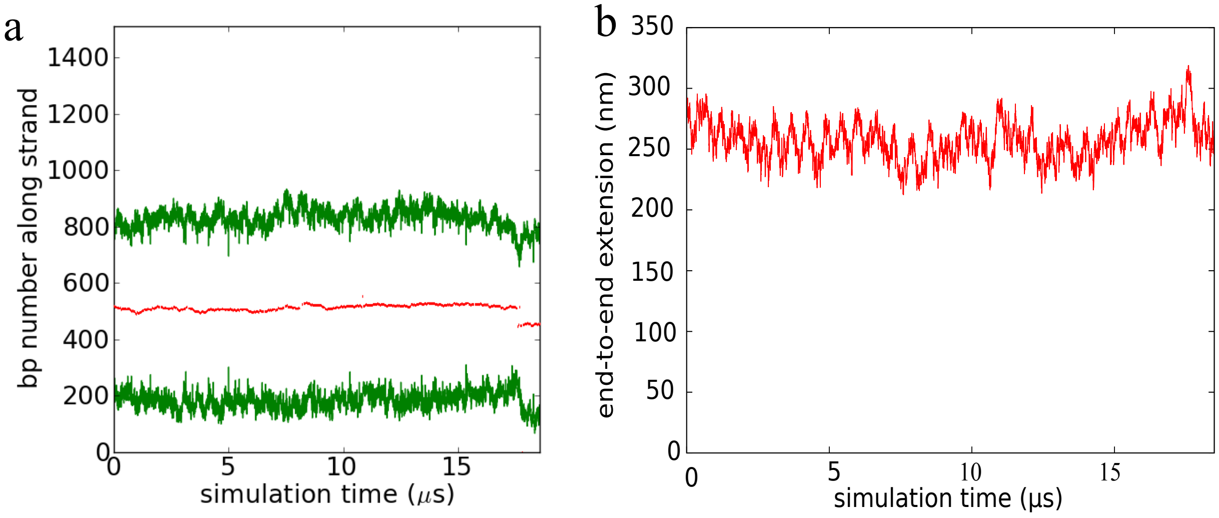

FIG. S12. (a) Position kymograph of a tip-bubble plectoneme at $L = 1500\,\mathrm{bp}$, for $\sigma = -0.08$ and $F = 2.3\,\mathrm{pN}$, showing stability of the structure on a μs time scale. (b) End-to-end extension kymograph for the run shown in (a).

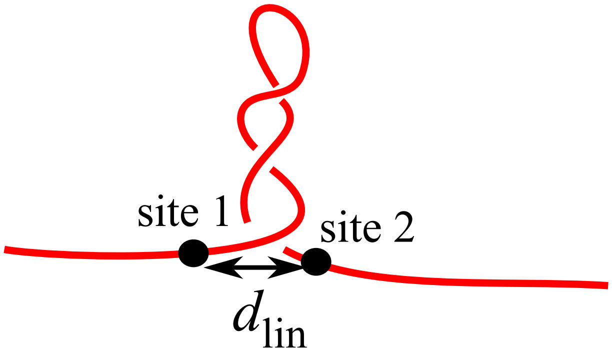

FIG. S13. In plectonemic regions, the direct spatial distance d_{lin} between two sites is shorter than the distance along the double-strand contour.

IV. DETERMINATION OF PLECTONEME POSITION, SIZE AND TIP BUBBLES

Plectoneme position and size

Plectoneme position and size are determined using an algorithm which relies on the fact that in plectonemes, the spatial distance d_{lin} between two sites on the molecule is smaller than their separation along the strand (Fig. S13). The algorithm only considers the midpoints between the centers of mass of corresponding bases on the single strands. It proceeds as follows:

- Start from strand end, loop over all midpoints
  - If any part of the remaining strand with a distance of more than N_c bp along the contour has a distance $d_{\mathrm{lin}} < d^0_{\mathrm{lin}}$, record the current bp index as the beginning of a plectonemic region, if the beginning of a plectoneme has not yet been detected.
  - If $d_{\mathrm{lin}} > d^0_{\mathrm{lin}}$ and a plectoneme beginning has been detected before, record the current bp index as the end of a plectonemic region and continue searching with the next bp
- The plectoneme position is the mean between the bp indices of the beginning and end of a plectonemic region
- The plectoneme size is the difference between the bp indices of the beginning and end of a plectonemic region

We performed plectoneme detection using $d^0_{\mathrm{lin}} = 7.24\,\mathrm{nm}$ and $N_c = 40\,\mathrm{bp}$. The results are not very sensitive to the precise choice of these parameters, as long as $d^0_{\mathrm{lin}} < N_c r_{\mathrm{bp}}$, where $r_{\mathrm{bp}} \approx 0.34\,\mathrm{nm}$ is the approximate rise of one base pair. N_c hence imposes a cutoff on the minimum size of plectonemes that can be detected with the search algorithm. The parameter choice made here ensures that writhed bubbles are reliably not counted as a plectonemic state, thus avoiding false positive detections. The algorithm is able to detect multiple plectonemes along the DNA double strand. However, for the salt conditions and strand length used in this work, only one plectoneme occurs in the simulated system. Two simultaneous plectonemic regions were only detected transiently during initial formation of the plectoneme. In order to obtain a single-valued plectoneme coordinate in these rare cases, we only consider the largest plectoneme structure.

The plectoneme tip-bubble state

For all sequences used in this work, we observed co-localization of plectonemes and bubbles. In these configurations the plectoneme position and the position of the midpoint of the largest denaturation bubble in the system coincide within a margin M_c.
A plectoneme with a tip bubble is defined as follows:

- A denaturation bubble with size $l_b \geq 2$ bp exists in the system.
- A plectoneme is detected in the system using the algorithm described in Supplementary Sec. IV.
- The bubble midpoint and the plectoneme position are separated by less than M_c bp

The restriction to bubbles with size $l_b \geq 2$ bp was introduced in order to discard short-lived single base-pair denaturations, which can occur in the strand due to thermal noise.

Fig. S14 shows distributions exhibiting strong co-localization of bubble and plectoneme. For a large majority of configurations, the bubble-plectoneme distance is less than 20 bp. We therefore chose $M_c = 20$ bp for the detection of tip-bubble plectonemes.

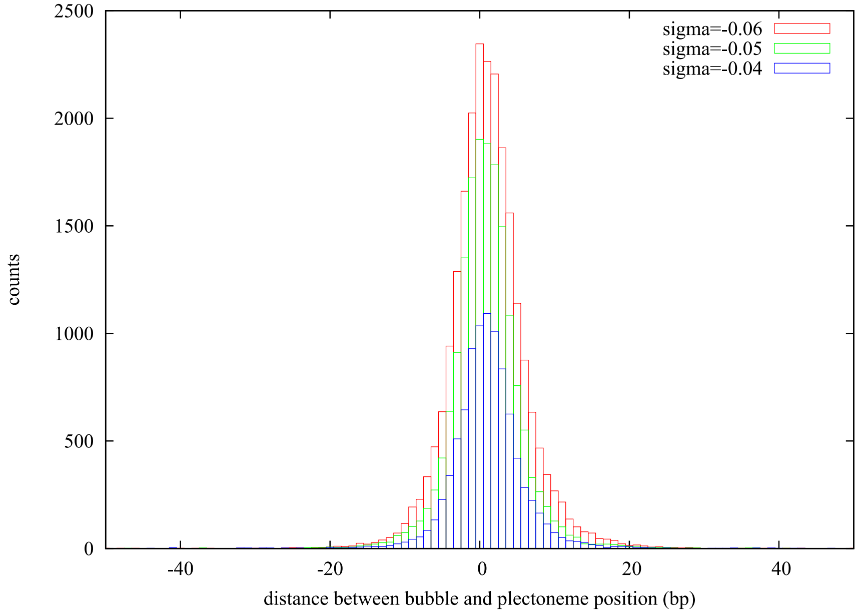

FIG. S14. Distribution for the distance between bubbles and plectoneme midpoints for simulations of a 600-bp system with the random sequence defined in Supplementary Sec. VIII, at different values of σ for $F = 1.27$ pN. Strong co-localization is observed.

V. INTERCONVERSION BETWEEN BUBBLE AND PLECTONEME SIZE

Bubbles in the end loop tend to shorten plectonemes due to three effects. Firstly, a denaturation in the end loop constitutes a defect which allows the formation of a smaller, more tightly wound tip of the plectoneme structure, as shown in Fig. 2a of the main text. This causes an extension ΔL of the overall system. Secondly, denaturation bubbles naturally possess very small twist. Thirdly, denatured single strands can twist back on themselves in a negative way, thus even leading to a negative twist contribution. As the overall linking difference ΔLk of the system is conserved according to the relation $\Delta Lk = \Delta Tw + \Delta Wr$ [8–10] (see Supplementary Sec. I), this increase in ΔTw changes the system's writhe component and thus the average plectoneme size. Fig. S15 shows free-energy landscapes of the system as a function of bubble and plectoneme size for different values of F (see also Figs. 2b and 2c of the main text). In a 600-bp strand, due to thermal noise, very short-lived denaturations of small size can occur in a duplex at $T = 300\,\mathrm{K}$. In order to separate out this contribution, bubbles with a size up of 1 or 2 bp were only taken into account if they were co-localized with a plectoneme.

Fig. S15 shows that after denaturation of the first two base pairs and consequent shape change of the end-loop, the size of plectonemes and bubbles can be interconverted in an approximately linear fashion, as seen also in Figs. 2b and 2c of the main text. For this system, the initial formation of a 2-bp tip bubble causes a decrease in plectoneme size by roughly 100 bp. Assuming that the equivalent plectoneme size is converted into extended strand length aligned with F and neglecting changes in bending energy, the free energy gain due to this extension is $\Delta G = F\Delta L \approx 1.27\,\mathrm{pN} \cdot 100 \cdot 0.34\,\mathrm{nm} \approx 10 k_B T$ for $T = 300\,\mathrm{K}$. This free energy gain is on the same order of magnitude as the cost of forming a small double strand denaturation in oxDNA.

Fig. S16 shows free energy profiles as a function of bubble size, corresponding to projections of the 2-dimensional free energy landscapes of Fig. S15 onto the x-axis. At $F \approx 1.5\,\mathrm{pN}$, a 2- bp tip bubble denaturation starts to become favourable. This small initial denaturation is particularly stable, as it enables kinking and therefore tighter winding of the end-loop, as shown in Fig. 2a of the main text. As the force increases, it becomes more favourable to grow larger bubbles, as can be seen in the free-energy plots in Figs. S15 and S16. For large enough force, the plectoneme disappears, and the dominant states are extended bubble states.

The thermodynamics of the crossover between tip-bubble plectonemes and extended bubbles is illustrated in Fig. S17, where the projection of Fig. S16 is separated into contributions due to plectonemes, and contributions due to bubble states without plectonemes. As discussed in Supplementary Sec. IV, the classification of tip-bubble plectonemes of small size somewhat depends on the cutoff of our plectoneme detection algorithm. However, as the transition from tip-bubble plectonemes to bubbles is fairly narrow (cf. Fig. 1b of the main text and Supplementary Sec. VII), this is not expected to significantly affect the force at which the crossover occurs. Fig. S17 clearly shows how, with increasing force, the system transitions from a regime with mainly tip bubbles, to a regime with mainly extended bubble states.

At parameter values where extended bubbles and tip-bubble plectonemes can coexist, local kinks induced by bubbles provide a preferred site for re-nucleation of a plectoneme. Structures and a corresponding kymograph are shown in Fig. S18 to illustrate the crossover dynamics between bubbles and plectonemes for $\sigma = -0.09$ and $F = 2.3$pN. The average size of both bubbles and plectoneme structures as a function of F at $\sigma = -0.08$ is shown in Fig. S19.

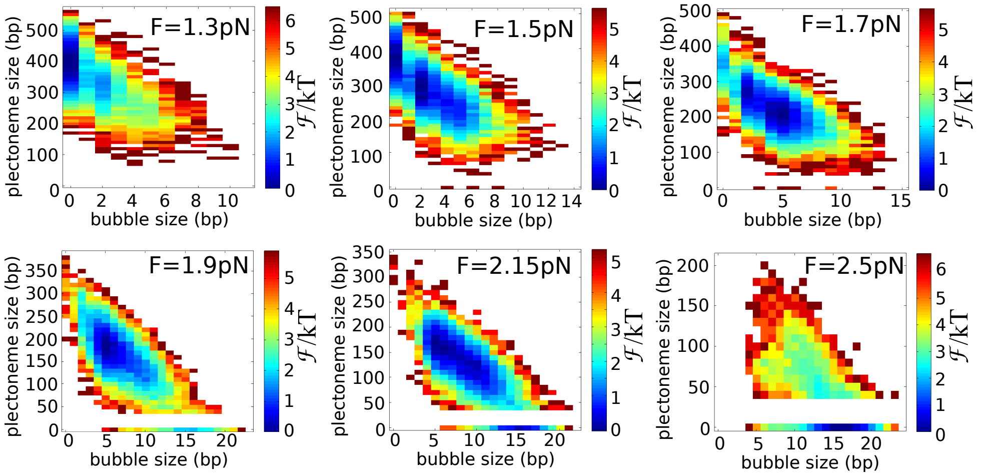

FIG. S15. Free energy $\mathcal{F}$ as a function of bubble and plectoneme size at $\sigma = -0.08$ and different forces. A negative linear correlation of bubble and plectoneme size is observed after forming a kink-like tip-bubble defect of 1 to 2 bp. The landscapes are obtained using the average-base parametrization of oxDNA.

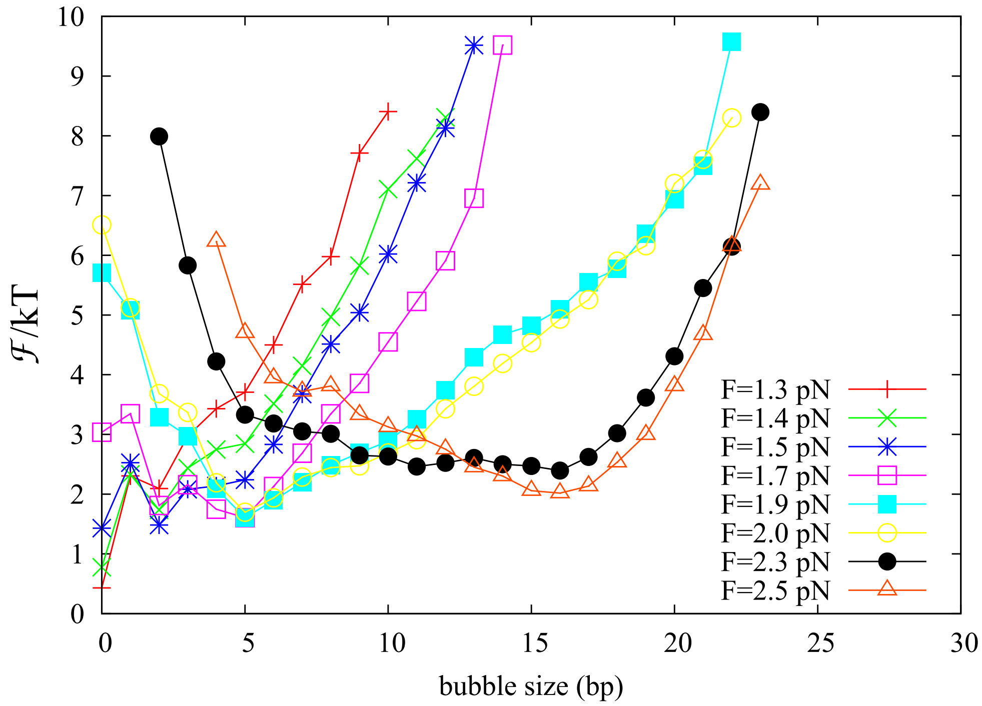

FIG. S16. Profiles of free energy $\mathcal{F}$ at $\sigma = -0.08$ and different values of F as a function of bubble size, corresponding to projections of the 2-dimensional free energy landscapes of Fig. S15 onto the x-axis. Note the stability of 2-bp bubbles at low force, due to free energy gains from initial end-loop rearrangement. For forces $F \gtrsim 2.3$ pN, extended bubble states are more stable than plectonemic states, see also Fig. S17.

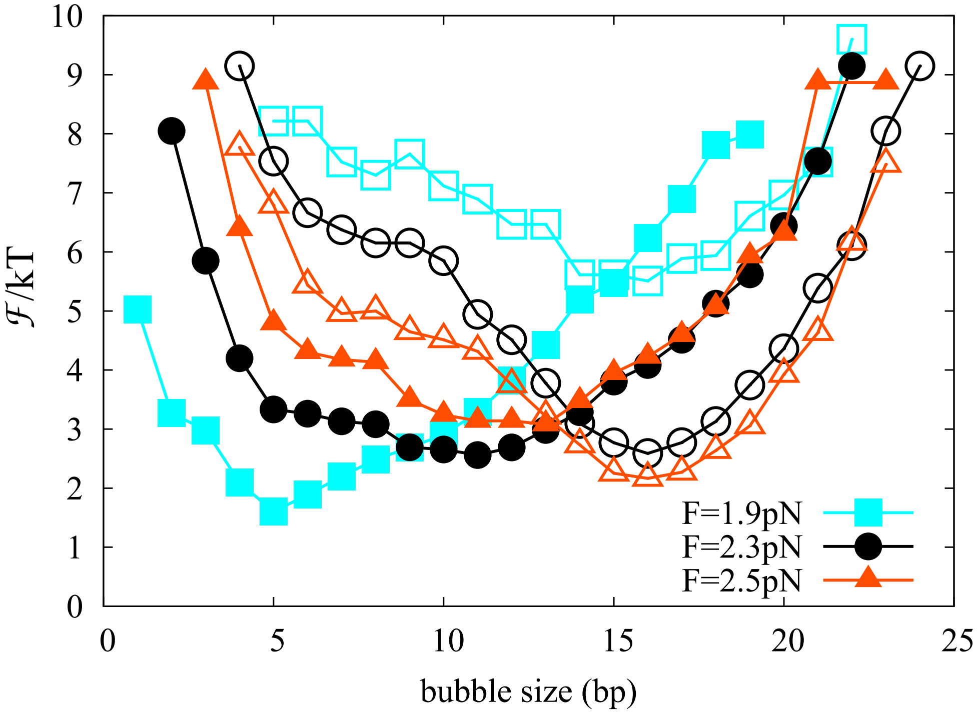

FIG. S17. Plots of free energy $\mathcal{F}$ that illustrate the thermodynamics of the crossover between tip bubbles and extended bubbles. The left curves (solid symbols) show the free energy of tip-bubble plectonemes at a particular size, while the right curves show the free energy of extended bubbles (open symbols) without a plectoneme. At small values of F, the tip bubble is more stable than the extended bubble. At $F \approx 2.3\,\mathrm{pN}$, the extended bubble population has a free energy roughly equal to the tip-bubble plectoneme, indicating that above this force, extended bubbles become more favourable, as can be seen for the $F = 2.5\,\mathrm{pN}$ curves.

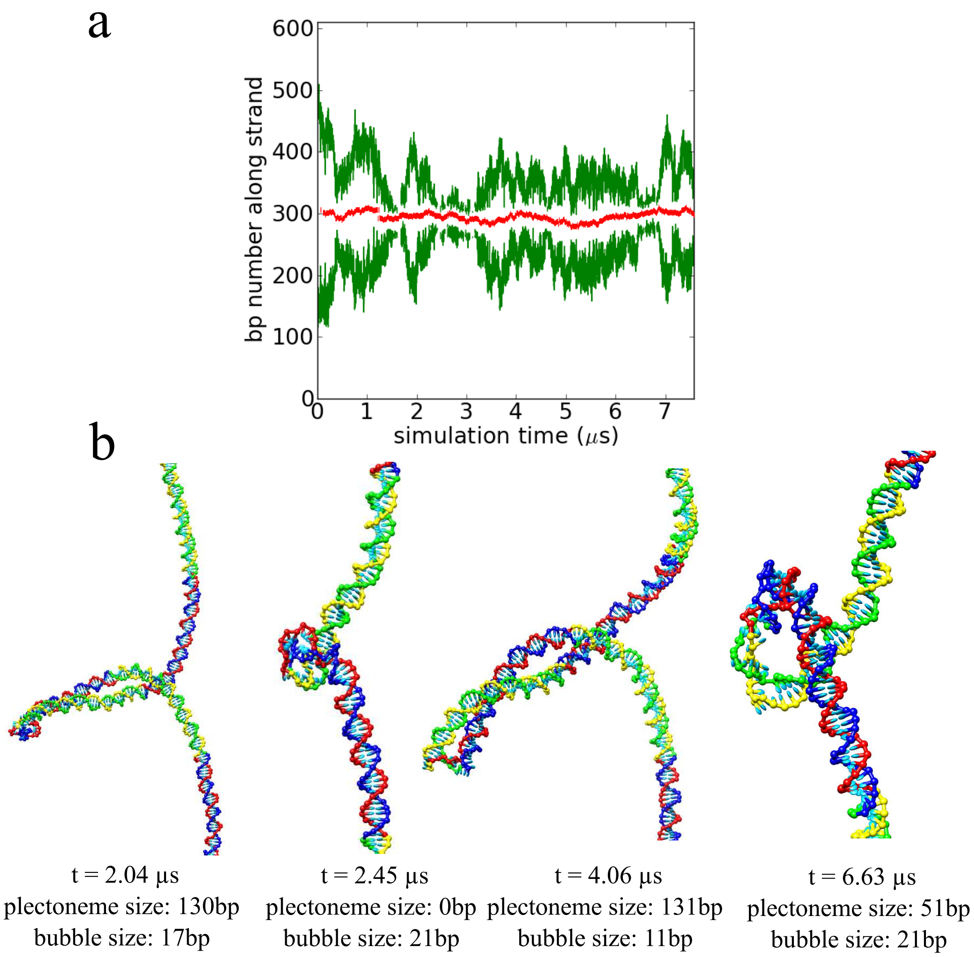

FIG. S18. Interconversion between bubbles and plectonemes at $\sigma = -0.09$ and $F = 2.3\,\text{pN}$. Different colouring of parts of the double strand is to facilitate comparison between structures. (a) Position kymograph of plectoneme boundaries (green) and bubble centre (red). (b) Example configurations taken from trajectory shown in (a) at the times indicated.

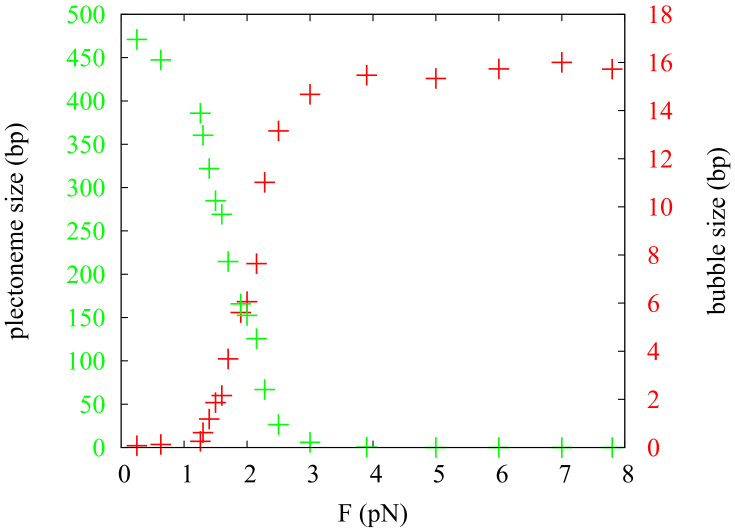

FIG. S19. Mean sizes of plectoneme (green) and denaturation bubble (red) as a function of applied stretching force F at $\sigma = -0.08$.

VI. DISPLACEMENT BEHAVIOUR OF PLECTONEMES

Diffusion

Fig. S20 shows the mean square displacement of plectoneme structures with and without tip bubbles in a 600-bp system, using the random sequence described in Supplementary Sec. VIII for $\sigma = -0.04$, $\sigma = -0.05$ and $\sigma = -0.06$, and a stretching force $F = 1.27\,\text{pN}$. The data was extracted from sections of trajectories of a total simulated time of $140\,\mu\text{s}$ for each value of σ. On a μs time scale, the mean-square displacements (MSD) $\langle (d(t))^2 \rangle$ of the positions of plectonemes without tip bubbles show an approximately linear behaviour in time, indicating diffusive motion of these structures. In contrast, tip-bubble plectonemes do not show any signature of MSD on that time scale. The diffusion coefficient D and the MSD are related by $\langle (d(t))^2 \rangle = 2Dt$, allowing determination of D from linear fits to the data shown in Fig. S20. The determined diffusion constant for unpinned structures decreases as σ becomes more negative because of the larger plectonemes that form at larger $|\sigma|$. The numerical values of D determined from the fitting procedure are given in Table S1.

An experimental observation of reduction in plectoneme diffusion with force under positive supercoiling was reported in Ref. 27. The diffusion constants obtained in this section are much higher than those measured in Ref. 27. There are several reasons for this difference. Firstly, at a strand length of 600 bp, even the highest superhelical density used to study diffusive motion, $|\sigma| = 0.06$, corresponds to a linking difference of $\Delta Lk \approx 3.5$. Thus, plectonemes of at most 3-4 double strand self-crossings are expected. In practice, plectoneme structures are slightly shorter, due to positive values of $\langle Tw \rangle$. In contrast to this, linking differences $\Delta Lk > 40$ were imposed in Ref. 27, leading to much larger plectoneme structures which are expected to diffuse far more slowly than the simulated structures. Secondly, a high effective monomer diffusion coefficient $D_{\text{sim}} = 6 \times 10^{-7}\,\text{m}^2\text{s}^{-1}$ was chosen in simulations for this work. This is a common choice in coarse-grained models, and increases the efficiency of sampling slow processes. From measurements of diffusive motion of DNA single strands, we estimate these effects to speed up the simulations by up to two orders of magnitude in simulated time [6]. Finally, underlying free-energy landscapes tend to be smoothed out in coarse-grained models, leading to accelerated motion of the simulated diffusion over barriers compared to the experimental system (see e.g. Ref. 28). The time scale used in our integrator is set by the mass, length and energy scales used, which determine the frequencies of intra-molecular vibration modes. Coarse-graining may affect differently processes such as the overall bending mode of the double strand, which are important for strand reptation, making it difficult to define a homogeneous time scale for all these processes. Therefore, it is generally safer to compare the relative time scale of two processes rather than their absolute duration.

Although a reduction in diffusion is seen in Ref. 27 with increasing applied force, the forces probed are lower than those at which tip-bubbles are seen for positive supercoiling in oxDNA. There are several possible explanations for this. Increasing the force is generally expected to lead to a slowdown of diffusion via reptation, due to tightening of the plectoneme structure [29]. This effect is illustrated in Fig. 3 a and b of the main text. Additionally, sequence-dependent mechanical properties of the strand beyond denaturation may come into play. Hence, it may be the case that the data in the specific experimental system of Ref. 27 can be explained without the formation of plectoneme bubbles. In that case, plectoneme bubbles would be expected to cause an additional subsequent slowdown of diffusion at higher forces. To directly demonstrate the role of tip-bubbles in experiment, it would be necessary to resolve the position of double-strand denaturations and plectonemes at the same time. However, it may be possible to indirectly measure their influence by showing that plectonemes are localised to AT rich regions.

σ	D (kbp^{2}s^{-1})	mean plectoneme size (bp)
-0.04	1.2×10^4	161
-0.05	5.1×10^3	218
-0.06	3.9×10^3	272

TABLE S1. Unpinned Diffusion constants obtained for different superhelical densities at $F = 1.27\,\text{pN}$ from the linear fits shown in Fig. S20.

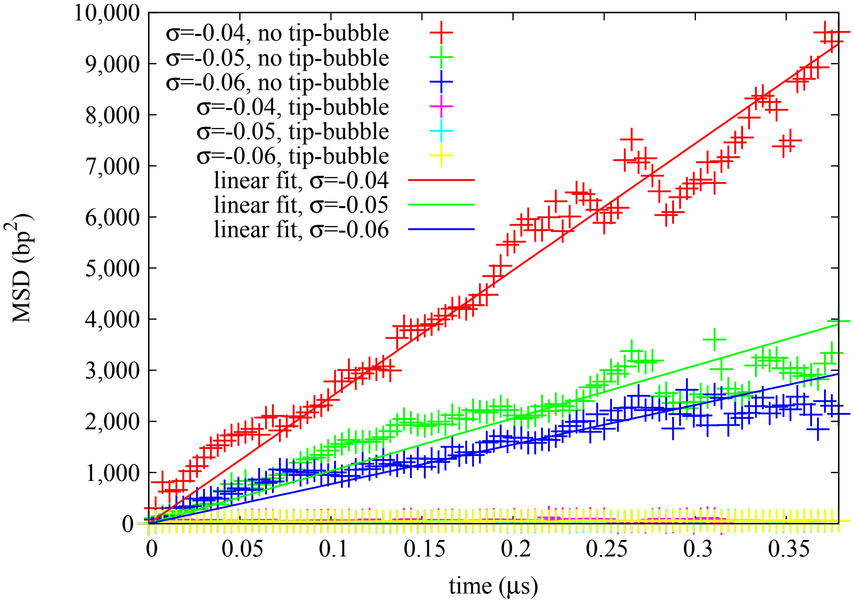

FIG. S20. Mean square displacement of pinned and unpinned plectonemes at different superhelical densities and $F = 1.27\,\text{pN}$. While tip-bubble plectonemes are pinned on a μs timescale, plectonemes without tip bubble exhibit significant diffusion.

Hopping

We observed long-range displacement of plectonemes by "hopping". In 21 independent simulations of a 600-bp system at $F = 1.27\,\text{pN}$ and $\sigma = -0.04$ run for a total simulation time of 148 μs, we observed 4 hopping events which involved rapid displacement of the plectoneme center position over a distance of more than 100 bp. In all cases, the plectoneme in the initial location fully unformed, and then quickly re-formed at a distant site (see Fig. S21 for a detailed plectoneme position kymograph and strand structures). Coexistence of two plectonemes was not observed in equilibrium, as expected at the high-salt conditions used in oxDNA [27, 30]. Due to the absence of equilibrium multi-plectoneme states in the 600-bp system, we observe hopping only close to the critical buckling superhelical density σ_b, where the system has a non-zero probability to dissolve a plectoneme and return to the extended state.

However, for DNA strands of length 1500 bp, we did observe long-range writhe exchange between two simultaneously present plectonemes in an out-of-equilibrium situation. Simulations were started from a linear, homogeneously undertwisted double strand at $\sigma = -0.08$ and $F = 2.3\,\text{pN}$. A typical example of such a run is shown in Fig. S22, where the kymograph initially shows the simultaneous presence of two plectonemes. Coexistence is followed by the disappearance of the smaller plectoneme, mediated by long-range transport of its writhe to the larger plectoneme,which then stays stable for a long time.

Van Loenhout *et al.* [27] found a similar long-range displacement behaviour over distances of up to 15 kbp at $F = 0.8\,\text{pN}$ and $\sigma \approx +0.04$. Some hopping events observed in the experiments of Ref. 27 showed immediate dissolution of the initial plectoneme, and re-nucleation at a distant site, which is reminiscent of the equilibrium mechanism described above. However, the experiments are performed at ambient conditions that permit stable coexistence of multiple plectonemes, so that the mechanism reminiscent of the one we observe in non-equilibrium situations is likely to be present as well.

A more detailed treatment of plectoneme hopping in strands of different lengths is an interesting open problem for further study.

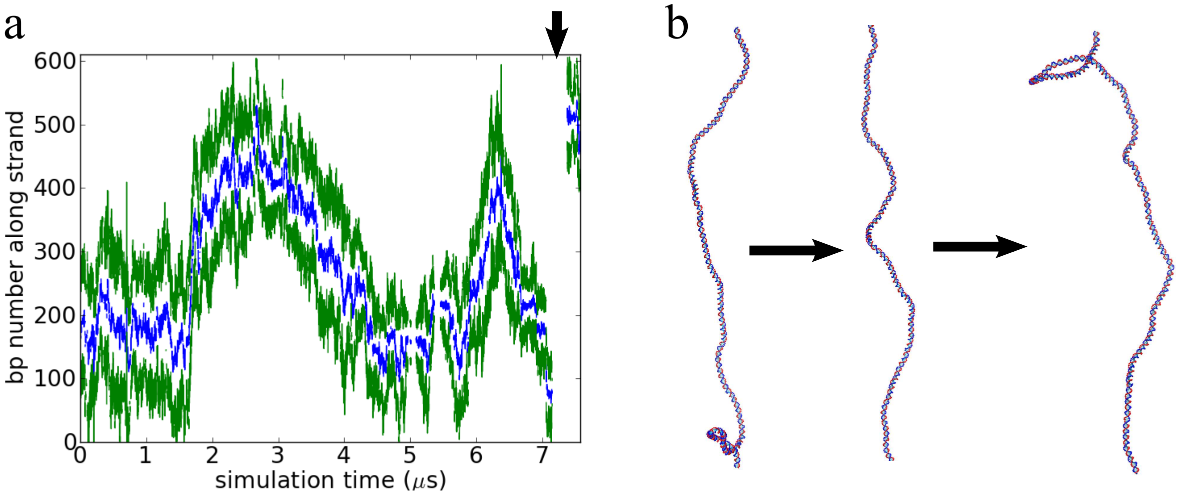

FIG. S21. Hopping event observed in a simulation at $\sigma = -0.04$ and $F = 1.27\,\mathrm{pN}$: (a) Kymograph of plectoneme boundaries (green lines) and centre position (blue line) showing a hopping event at the time marked by a black arrow. (b) Structure of the DNA strand close to the hopping event at $t = 7.09\,\mu\mathrm{s}$ (left), $t = 7.34\,\mu\mathrm{s}$ (middle) and $t = 7.39\,\mu\mathrm{s}$ (right).

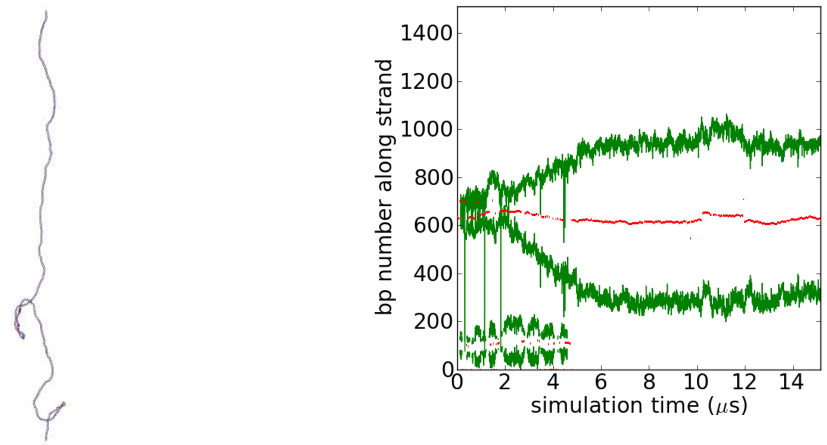

FIG. S22. Coexisting plectonemes in an out-of-equilibrium run started from a homogeneously underwound linear double strand of length 1500 bp at $\sigma = -0.08$ and $F = 2.0\,\mathrm{pN}$, as shown in the Supplementary Movie. left: Molecule configuration showing two coexisting plectonemes. right: Kymograph of two plectoneme sites. Ultimately, the smaller plectoneme dissolves and a single, large plectoneme is formed.

VII. POPULATION FREQUENCIES OF DIFFERENT DNA STRUCTURES

This section shows the population frequencies of bubbles, plectonemes and tip-bubble plectonemes as a function of F for different fixed values of σ, as in Fig. 1b of the main paper. The pictures correspond to "cuts" along axes parallel to the y-axis of the state diagram (cf. Fig. 1c of the main paper). We used a population of 40% in these plots to define the boundaries in the state-diagram shown in Fig. 1c of the main paper.

For $\sigma < 0$, there is a clear crossover from pure plectonemes to tip-bubble plectonemes and then to pure bubbles as a function of force. For $\sigma > 0$, a broader crossover to the tip-bubble regime occurs. At low values of $|\sigma|$, the recognition of small plectonemes somewhat depends on the cutoff values chosen in the plectoneme detection algorithm, as described in Supplementary Sec. IV. As the transitions in this region of the state diagram are narrow as a function of F and σ, this has only a small effect on the positions of state boundaries.

Note that as long as σ is large enough to allow stable tip bubbles, the transition from plectonemes to tip bubbles appears to be at a very similar force for different σ. The reason for this is that the crossover is mainly determined by a change in the end-loop structure, rather than in the rest of the plectoneme, which grows for increasing σ. Similar arguments may explain the observation that the transition from tip bubbles to extended bubbles also happens at the same force for different negative values of σ.

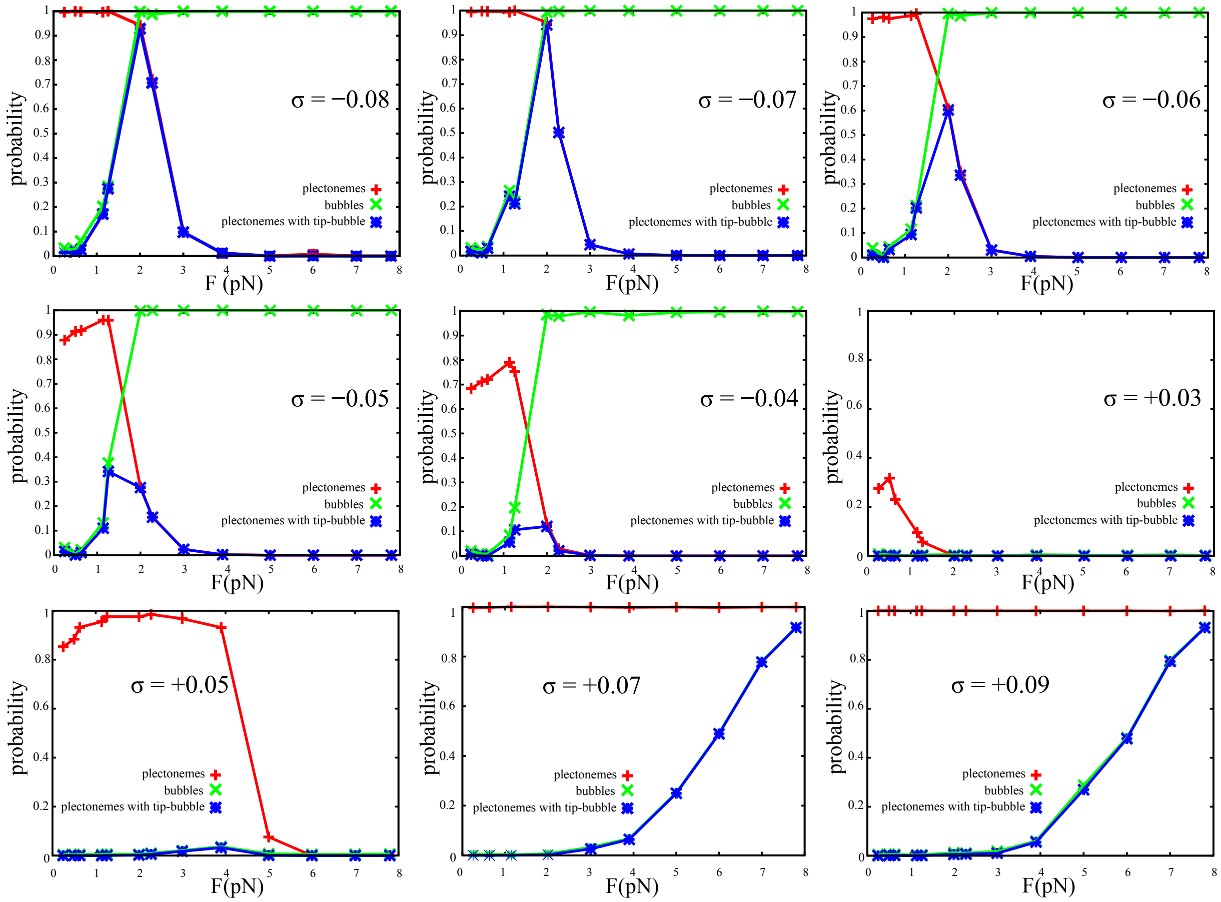

FIG. S23. Population diagrams as a function of force for different imposed values of σ, analogous to Fig. 1b of the main paper. For $\sigma < 0$, note the occurrence of a tip-bubble regime for intermediate values of F. At $\sigma > 0$, note the wide crossover to tip-bubble plectonemes for increasing F. No pure bubble regime exists for $\sigma > 0$ at the force values used here. However, other forms of DNA may occur at sufficiently larger force.

VIII. SEQUENCE-DEPENDENT PROPERTIES

Underlying base sequences

In this work, we studied sequence-dependent properties of plectonemes using the sequence-dependent parametrization of oxDNA [13]. The sequences we used in the study of sequence-dependent behaviour are:

Random sequence:

```
5'-AGAGTACTTAGGCTTGACGATTTCGCGCCTGAACTTCTGATAACTCAGTCTGAGAGACTAAGTTGACGTTCTATCCATCATCA
GGTGGGCTCAGAGATTGTGCGGCAGACTTAAGTGTAGTACCAGCTGCTGGTCAATTTGATCTATGCTGATCCGCTCGGAACGGGCC
GTGAAAGAAGTACTCTCGCCTATAGAACGGTTAGTGCTACGACTTTTGCGCGACACAATGTGGTAGTTATCTTCTGTTTTCCTGAA
TAGTGAGCCTACCAGAAGAGGCCACCGACAAATCTGATGAGATAGACGGGAACACGGTTTGCGGAGCCTCTGAAACGCTTGTTTAT
GAGCAAGAGAGGTGCGGTGGGTATGACCGCCGTAGAAGTACCGTATTCTTCCGGGCTCGGTGGCAATGAACACTTAAGGGGCCGAC
ACATTCTGAAGTCAATCGATGGACGGACCTCAACCGTGCACCCTTCTATATACGTGTGGCTAGGATACTCTAGCGTTTACCCGCCG
TCTTCCACGATGCCGAATATAAGCCGAGGATAAAGGTGCAGACAAATATCAGGCTTCGCAGTTGTGTAACTTCCTGTATTGTTGTG
C-3'
```

This sequence was chosen at random, with a 25% probability for each possible base identity.

Block-random sequence:

```
5'-AGACTCGACCGACCCGCGAGATCGGCTCCAGTCTCTGCGCCAAGTGCCGTTGCCCCGTCTCGTGGGCCGGTCGGTGAACCTTC
ATACGGTGGGAGTCGCTGAGGCCGTCCCATTTGTACCCATCGAACTCTTATTTTGTATTTTTTGGACATCCTCAGCTAACCACACG
AGCCAAGCTATAGATCAGATTTGGGTATTCGGCGATCTTTCTAATCAACTGTATCCGATGCTATACAGATACTTTATTCTAAGGCG
GTCCGCGATGCGCCCAGTCCGTTGACCGGGCGAGTCATGTCAGAGTCGGCAATTATCGGGCACGTCGCCGGGGTGATACGTCCCTG
TGTCACTAGGCATAGGTCGTAACATATGATTATATATACTTTCCACTTTATGTATATCATTTGCAAGTTAGACATAATAAGGATAT
ATAATATAAGAATCTCTTCACCTCTAAAGTGAGTGATTGGAATATAAGTATTTGCGCCACTACCCGGCCGAAAGCCCGCGGCTCCT
CGCGGGTAGGTTGCCGGGGACCCGCGTGAAGAAAGGATGAAGCACCCGGACGCCCGCCTGCGAGTTGGCCACGGGCCCATAACGGC
G-3'
```

Above, stretches of 120 bp length are colored, with blue regions having a high GC content and red regions a high AT content. In an abbreviated form, the block-random sequence can be written as 5'-$S_1 W_1 S_2 W_2 S_3$-3'. The overall GC content of the block-random sequence is 52%. The GC contents of the individual 120 bp stretches is given in Table S2.

TABLE S2. GC-contents of 120 bp stretches in the block-random sequence

stretch	GC content	AT content
S_1	65.0%	35.0%
W_1	39.2%	60.8%
S_2	60.0%	40.0%
W_2	24.2%	75.8%
S_3	71.7%	28.3%

plectoneme position distribution in average-base model

As reported in Fig. 3c of the main paper, the distribution of plectonemes exhibits a marked sequence dependence which is induced by the sequence-dependent enthalpic cost of forming tip-bubble denaturations. In order to distinguish this sequence-dependent effect from the generic localization behaviour, we also ran simulations under the same stretching force and undertwist ($\sigma = -0.06$ and $F = 1.27\,\mathrm{pN}$) for the average-base parametrization of oxDNA. Comparison between the two sequences studied and the average-base parametrization are shown in Figs. S24 and S25. Due to the repulsion plane boundary described in Supplementary Sec. I and finite plectoneme size, the probability for the position of plectoneme midpoints decreases towards the strand ends. A clear influence of the sequence on plectoneme position is shown in Figs. S24 and S25.

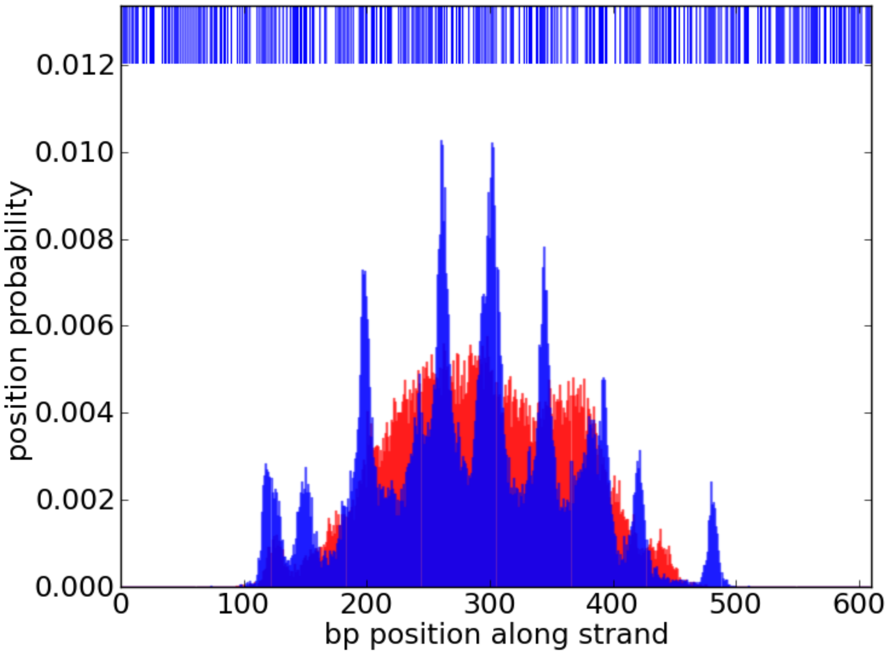

FIG. S24. Plectoneme position distribution for the random sequence using the sequence-dependent (blue) and average-base (red) parametrization at $\sigma = -0.06$ and $F = 1.27\,\mathrm{pN}$. The sequence is given in the upper part of the figure, where a blue line indicates an AT base-pair. Mean plectoneme size is 260 bp for the average-base parametrization and 195 bp for the random sequence.

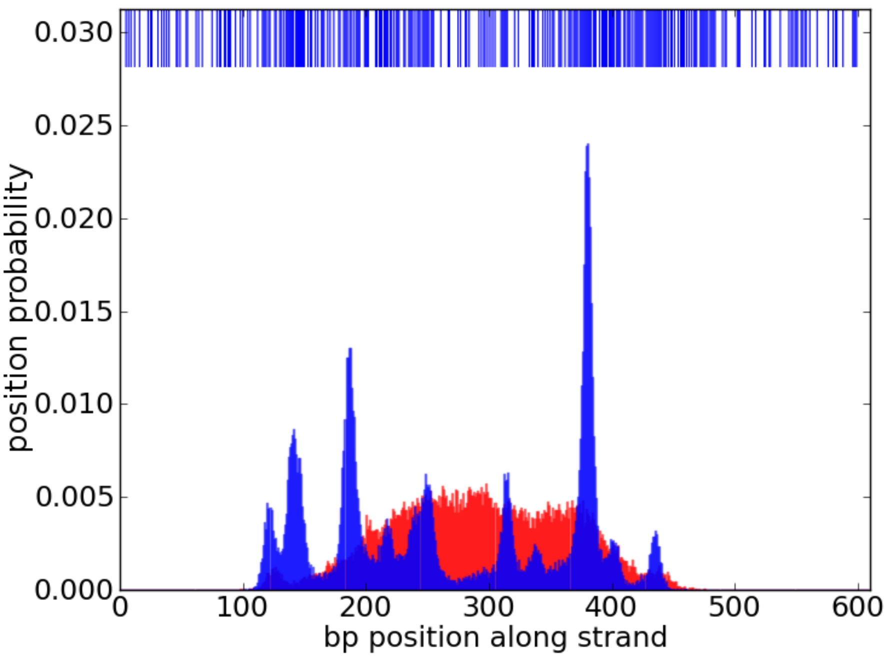

FIG. S25. Plectoneme position distribution for the block-random sequence using the sequence-dependent (blue) and average-base (red) parametrization at σ=-0.06 and F = 1.27 pN. The sequence is given in the upper part of the figure, where a blue line indicates an AT base-pair. Mean plectoneme size is 260 bp for the average-base parametrization and 137 bp for the block-random sequence.

Sequence effects in extension curves

In Fig. S26 we compare extension "hat curves" obtained for both the average-base and sequence-dependent parametrization of oxDNA. Sequence-dependent simulations were performed for the random sequence given above. While outside the bubble regime, results show no significant difference, the sequence-dependent model exhibits a systematically larger extension when forces become big enough to allow denaturation. This indicates that denaturation occurs more easily in the sequence-dependent case than for the average-base parametrization.

Such behaviour might be expected, as weak AT base pairs represent preferred sites of base-pair breaking, making denaturation of the strand less enthalpically costly, while not affecting the bending energy cost much. From simple considerations of energy scaling for the competition between denaturation and plectoneme formation, the crossover force can be estimated to depend on the free energy of base pair breaking α as $F_{\rm char} \propto \alpha^2/B_0$ [23]. Hence, sequence-dependent differences in denaturation energy are expected to have a noticeable effect on strand extension properties. As denaturation bubbles can grow to significantly larger sizes than 1 bp, not only the overall AT content of the strand, but also the base distribution may influence extension properties (see also the previous discussion on the block random sequence). The detailed influence of sequence properties on strand extension behaviour represents an interesting problem for further study, which we are planning to address in the future.

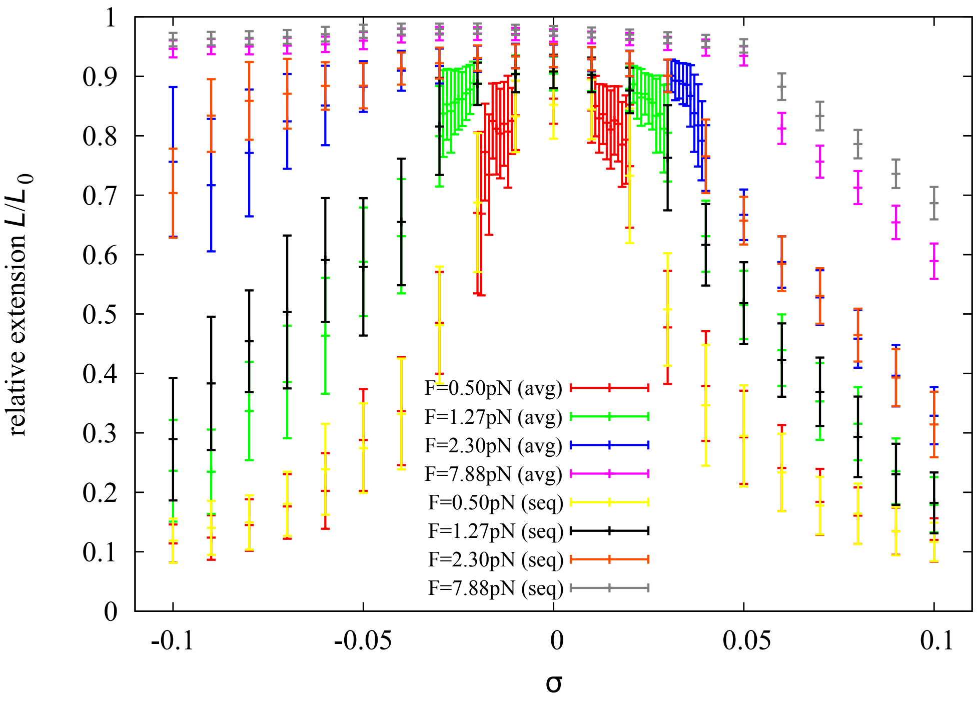

FIG. S26. Comparison of extension curves for the random sequence given above in the average-base and sequence-dependent parametrization of oxDNA. For parameter values for which tip-bubble plectonemes occur, sequence-dependent simulations show a systematically higher extension, as weak regions in the sequence provide preferred sites of base-pair breaking, thus enhancing overall strand denaturation. Error bars indicate thermal fluctuations in the end-to-end distance, rather than sampling uncertainties.

IX. COMPARISON TO OTHER MODELLING APPROACHES

DNA under superhelical stress has been modelled using various approaches on different scales of resolution. Here, we briefly compare oxDNA to other models applicable to similar problems.

On a continuum level DNA is commonly described as a semi-flexible polymer. These models provide an efficient representation of DNA on large length scales, and have been widely used to study supercoiled DNA [31]. Continuum models have also been used to study the phase behaviour of DNA plectonemes and other states deviating from the linear B-helical configuration [19, 21, 32]. These models have been very successful at describing the effective mechanical behaviour of DNA on larger length scales. oxDNA captures the elastic behaviour of DNA, but also naturally has access to the physics of base-pair formation, which is important for the tip-bubble plectoneme regime we study in this work. It would be very interesting to refine continuum models by using oxDNA, to allow the direct calculation of tip-bubble plectonemes for positive and negative supercoiling.

To study the statistical properties of DNA denaturation, an important model has been developed by Benham and co-workers [25, 33, 34]. It is a thermodynamic model based on considering the effective free energy contributions of denatured and non-denatured strand regions. Its simplicity allows rapid calculations of the statistical denaturation properties of supercoiled DNA on a genomic level [35]. Among other applications, it has been used successfully to describe sizes and locations of denaturation bubbles in circular DNA [36]. However, the model does not explicitly account for DNA structure beyond denaturation, or provide microscopic information on strand dynamics, processes which are important for the tip-bubble plectoneme regime. It would be interesting to see if a similar thermodynamic model can be adapted to predict the distribution of locations and sizes of tip-bubble plectonemes.

On a much more structurally detailed level, DNA response to supercoiling has been studied in atomistic simulations [37–39]. As these simulations are computationally expensive, they have only recently been used to access extended DNA structures under superhelical stress. Atomistic simulations have been able to study local structural defects in small supercoiled minicircles, in particular microscopic properties of kinking at denaturations [40, 41]. Recent experiments showed good agreement with the geometric picture of minicircles these atomistic simulations provide [42]. Due to their computational expense, the timescales of these simulations are comparatively short, making the extraction of equilibrium properties difficult, in particular for writhed structures. For this reason, modelling of the properties of long DNA strands under twist and stretching force, as performed in this work, is currently outside the reach of fully atomistic models. It may be interesting to prepare locally bent configurations in a plectonemic state, and use atomistic simulations to observe the local detailed configuration of a tip bubble.

Coarse-grained models, which combine structural information on a higher level of resolution with the possibility to measure thermodynamically averaged quantities, are needed to fill the gap between thermodynamic models and continuum models on the one hand and atomistic models on the other hand. In Refs. 7, 12 and 13, we review a number of other coarse-grained DNA models, although the interplay between twist, writhe and denaturation has not received much attention. One model that has been used to study the interplay between denaturation and writhe is the coarse-grained model by Mielke *et al.* [43]. In this approach, a single coarse-grained unit represents 3 nucleotides. The authors considered 141-bp minicircles, which they twisted in a dynamical fashion, up to $\sigma = -0.47$, a very high superhelical density. For a few runs, the authors saw evidence that initial denaturation occurred at weak points in the sequence, near the loops of the writhed figure-of-8 structures that would form prior to denaturation. As such, they saw tantalising evidence of coupled writhe and denaturation. As the model of Ref. 43 does not allow reversible base-pair denaturation, its applicability is limited to non-equilibrium situations. It would be interesting to study the same system with oxDNA to see if this result for minicircles is robust in equilibrium. One can imagine quite a number of other DNA configurations where the coupling of twist, writhe and denaturation may be interesting to study with oxDNA.

[1] Russo, J., Tartaglia, P. & Sciortino, F. Reversible gels of patchy particles: role of the valence. *J. Chem. Phys.* **131**, 014504 (2009).
[2] Padding, J. T. & Louis, A. A. Hydrodynamic interactions and Brownian forces in colloidal suspensions: coarse-graining over time and length scales. *Phys. Rev. E* **74**, 031402 (2006).
[3] Louis, A. A. Coarse-graining dynamics by telescoping down time-scales: comment for Faraday FD144. *Faraday Discuss.* **133**, 323 (2010).
[4] Murtola, T., Bunker, A., Vattulainen, I., Deserno, M. & Karttunen, M. Multiscale modeling of emergent materials: biological and soft matter. *Phys. Chem. Chem. Phys.* **11**, 1869–1892 (2009).
[5] Romano, F., Chakraborty, D., Doye, J. P. K., Ouldridge, T. E. & Louis, A. A. Coarse-grained simulations of DNA overstretching. *J. Chem. Phys.* **138**, 085101 (2013).
[6] Ouldridge, T. E., Šulc, P., Romano, F., Doye, J. P. K. & Louis, A. A. DNA hybridization kinetics: zippering, internal displacement and sequence dependence. *Nucleic Acids Res.* **41**, 8886–8895 (2013).
[7] Doye, J. P. K. *et al.* Coarse-graining DNA for simulations of DNA nanotechnology. *Phys. Chem. Chem. Phys.* **15**, 20395–20414 (2013).
[8] Călugăreanu, G. L'intgrale de Gauss et l'analyse des Noeuds tridimensionnels. *Rev. Roum. Math. Pures et Appl.* **4**, 5–20 (1959).
[9] White, J. H. Self-linking and the Gauss Integral in Higher Dimensions. *Am. J. Math.* **91**, 693–728 (1969).
[10] Fuller, F. B. The writhing number of a space curve. *Proc. Natl. Acad. Sci. USA* **68**, 815–819 (1971).
[11] Matek, C., Ouldridge, T. E., Levy, A., Doye, J. P. K. & Louis, A. A. DNA cruciform arms nucleate through a correlated but asynchronous cooperative mechanism. *J. Phys. Chem. B* **116**, 11616–11625 (2012).
[12] Ouldridge, T. E., Louis, A. A. & Doye, J. P. K. Structural, mechanical, and thermodynamic properties of a coarse-grained DNA model. *J. Chem. Phys.* **134**, 085101 (2011).
[13] Šulc, P. *et al.* Sequence-dependent thermodynamics of a coarse-grained DNA model. *J. Chem. Phys.* **137**, 135101 (2012).
[14] Strick, T. R., Allemand, J. F., Bensimon, D., Bensimon, A. & Croquette, V. The elasticity of a single supercoiled DNA molecule. *Science* **271**, 1835–1837 (1996).
[15] Brutzer, H., Luzzietti, N., Klaue, D. & Seidel, R. Energetics at the DNA supercoiling transition. *Biophys. J.* **98**, 1267–1276 (2010).
[16] Janssen, X. J. A. *et al.* Electromagnetic torque tweezers: a versatile approach for measurement of single-molecule twist and torque. *Nano Lett.* **12**, 3634–3639 (2012).
[17] Mosconi, F., Allemand, J. F., Bensimon, D. & Croquette, V. Measurement of the torque on a single stretched and twisted DNA using magnetic tweezers. *Phys. Rev. Lett.* **102**, 078301 (2009).
[18] Forth, S. *et al.* Abrupt buckling transition observed during the plectoneme formation of individual DNA molecules. *Phys. Rev. Lett.* **100**, 148301 (2008).
[19] Marko, J. F. & Neukirch, S. Competition between curls and plectonemes near the buckling transition of stretched supercoiled DNA. *Phys. Rev. E* **85**, 011908 (2012).
[20] Maffeo, C. *et al.* DNA-DNA interactions in tight supercoils are described by a small effective charge density. *Phys. Rev. Lett.* **105**, 158101 (2010).
[21] Neukirch, S. & Marko, J. F. Analytical description of extension, torque, and supercoiling radius of a stretched twisted DNA. *Phys. Rev. Lett.* **106**, 138104 (2011).
[22] Tempestini, A. *et al.* Magnetic tweezers measurements of the nanomechanical stability of DNA against denaturation at various conditions of pH and ionic strength. *Nucleic Acids Res.* **41**, 2009–2019 (2013).
[23] Salerno, D. *et al.* Single-molecule study of the DNA denaturation phase transition in the force-torsion space. *Phys. Rev. Lett.* **109**, 118303 (2012).
[24] Moroz, J. D. & Nelson, P. Torsional directed walks, entropic elasticity, and DNA twist stiffness. *Proc. Natl. Acad. Sci. USA* **94**, 14418–14422 (1997).
[25] Fye, R. & Benham, C. Exact method for numerically analyzing a model of local denaturation in superhelically stressed DNA. *Phys Rev E* **59** (1999).
[26] Daniels, B. C. & Sethna, J. P. Nucleation at the DNA supercoiling transition. *Phys. Rev. E* **83**, 041924 (2011).
[27] van Loenhout, M. T. J., de Grunt, M. V. & Dekker, C. Dynamics of DNA supercoils. *Science* **338**, 94–97 (2012).
[28] Hills, R. D., Lu, L. & Voth, G. A. Multiscale coarse-graining of the protein energy landscape. *PLoS Comput. Biol.* **6**, e1000827 (2010).
[29] de Gennes, P. G. Tight knots. *Macromolecules* **17**, 703–704 (1984).
[30] Emanuel, M., Lanzani, G. & Schiessel, H. Multi-plectoneme phase of double-stranded DNA under torsion. *Phys. Rev. E* **88**, 022706–1–20 (2013).
[31] Marko, J. F. & Siggia, E. D. Fluctuations and supercoiling of DNA. *Science* **265**, 506–508 (1994).
[32] Marko, J. F. & Neukirch, S. Global force-torque phase diagram for the DNA double helix: structural transitions, triple points, and collapsed plectonemes. *Phys. Rev. E* **88**, 062722 (2013).

[33] Benham, C. J. Energetics of the strand separation transition in superhelical dna. *J Mol Biol* **225**, 835–847 (1992).

[34] Bauer, W. R. & Benham, C. J. The free energy, enthalpy and entropy of native and of partially denatured closed circular dna. *J Mol Biol* **234**, 1184–1196 (1993). URL `http://dx.doi.org/10.1006/jmbi.1993.1669`.

[35] Strawbridge, E. M., Benson, G., Gelfand, Y. & Benham, C. J. The distribution of inverted repeat sequences in the Saccharomyces cerevisiae genome. *Curr. Genet.* **56**, 321–340 (2010).

[36] Jeon, J.-H., Adamcik, J., Dietler, G. & Metzler, R. Supercoiling induces denaturation bubbles in circular DNA. *Phys. Rev. Lett.* **105**, 208101 (2010).

[37] Randall, G. L., Zechiedrich, L. & Pettitt, B. M. In the absence of writhe, DNA relieves torsional stress with localized, sequence-dependent structural failure to preserve B-form. *Nucleic Acids Res.* **37**, 5568–5577 (2009).

[38] Kannan, S. & Zacharias, M. Simulation of DNA double-strand dissociation and formation during replica-exchange molecular dynamics simulations. *Phys. Chem. Chem. Phys.* **11**, 10589–10595 (2009).

[39] Harris, S. A. Modelling the biomechanical properties of DNA using computer simulation. *Phil. Trans. A* **364**, 3319–3334 (2006).

[40] Liverpool, T. B., Harris, S. A. & Laughton, C. A. Supercoiling and denaturation of DNA loops. *Phys. Rev. Lett.* **100**, 238103 (2008).

[41] Mitchell, J. S., Laughton, C. A. & Harris, S. A. Atomistic simulations reveal bubbles, kinks and wrinkles in supercoiled DNA. *Nucleic Acids Res.* **39**, 3928–3938 (2011).

[42] Lionberger, T. A. *et al.* Cooperative kinking at distant sites in mechanically stressed DNA. *Nucleic Acids Res* **39**, 9820–9832 (2011).

[43] Mielke, S. P., Grønbech-Jensen, N., Krishnan, V. V., Fink, W. H. & Benham, C. J. Brownian dynamics simulations of sequence-dependent duplex denaturation in dynamically superhelical DNA. *J. Chem. Phys.* **123**, 124911 (2005).
